# Effectiveness of Influenza Vaccines and Duration of Protection Against Hospitalisation in England: 2022/2023 and 2023/2024 Seasons

**DOI:** 10.1111/irv.70194

**Published:** 2025-11-27

**Authors:** Heather J. Whitaker, Freja C. M. Kirsebom, Katie Hassell, Catherine Quinot, Julia Stowe, Katja Hoschler, Maria Zambon, Nick J. Andrews, Conall H. Watson, Jamie Lopez Bernal

**Affiliations:** ^1^ Modelling Division UK Health Security Agency London UK; ^2^ Immunisation and Vaccine‐Preventable Diseases Division UK Health Security Agency London UK; ^3^ Virus Reference Unit UK Health Security Agency London UK

**Keywords:** adjuvanted influenza vaccine, cell‐based influenza vaccine, inactivated influenza vaccine, influenza, live attenuated influenza vaccine, recombinant influenza vaccine, test‐negative design, vaccine effectiveness

## Abstract

**Background:**

Linkage of national, healthcare data can provide greater resolution of vaccine effectiveness (VE) against influenza subtypes, as well as the relative effectiveness of different vaccine types and the effects of waning on VE. We present 2022/2023 and 2023/2024 end‐of‐season VE against hospitalisation with laboratory confirmed influenza for England.

**Methods:**

A test‐negative design was used to estimate VE against hospitalisation. Cases and controls were identified within national laboratory surveillance systems and linked to hospital admission data. Vaccine histories were obtained from England's vaccine register. We combined results on VE by vaccine type over the two seasons using a meta‐analysis.

**Results:**

One hundred thousand five hundred eighty‐one (28,565 positive) and 113,494 (21,243 positive) samples were eligible for inclusion in 2022/2023 and 2023/2024, respectively. The overall VE for children aged 2–17 years was 67% (95% CI, 63%–70%) in 2022/2023 and 56% (95% CI, 51%–60%) in 2023/2024. For adults aged 18–64, VE was 34% (95% CI, 30%–38%) in 2022/2023 and 38% (95% CI, 34%–42%) in 2023/2024. For adults aged 65+ years, VE was 25% (95% CI, 21%–29%) in 2022/2023 and 18% (95% CI, 14%–22%) in 2023/2024. We found no significant difference in VE by vaccine type. We found evidence of reduced VE against influenza A in adults 9 weeks or more post vaccination compared with 2–8 weeks post vaccination.

**Conclusions:**

Vaccine protection against influenza hospitalisation was seen in all age groups, with strong protection for children. Adult protection could be strengthened by vaccination closer to the time of expected influenza circulation. Linkage of routine healthcare data provided us with a sufficiently large data set to estimate differences in VE by vaccine type and timing after vaccination.

## 1 Introduction

The United Kingdom (UK) offers free influenza vaccination to all adults aged 65 years and older (65+), individuals aged 6 months to 64 years in clinical risk groups, pregnant women, health and social care workers, household contacts of immunocompromised persons, and carers. Since 2013, there has been an incremental rollout in England of a children's programme primarily using live attenuated influenza vaccine (LAIV) vaccination [1]. During the 2022/2023 campaign, vaccination was offered first to preschool and primary school‐aged children aged 2–11 and subsequently to secondary school‐aged children aged 11–15, prioritising 11–13 year olds. During the 2023/2024 campaign, vaccination was offered to all children aged 2–15 years. During three seasons from 2020/2021 to 2022/2023 amid the COVID‐19 pandemic, influenza vaccination was also offered to all adults aged 50–64 years in England [2, 3].

In 2022/2023, following two seasons of reduced circulation in the United Kingdom during the COVID‐19 pandemic, influenza returned to pre‐pandemic levels. Historically, low vaccine effectiveness (VE) against A(H3N2) influenza strains has been seen among older adults with several explanations hypothesised including immunosenescence, waning VE, antigenic mismatch, egg adaption of the A(H3N2) vaccine viruses and back‐boosting [4, 5]. Quadrivalent adjuvanted (aQIV; since 2018/2019), cell‐based (QIVc; since 2019/2020) and recombinant (QIVr; since 2021/2022) vaccines have been prioritised in the UK adult vaccine schedule with hopes of better VE than seen historically for standard‐dose nonadjuvanted egg‐based vaccines (QIVe).

Several reviews on the effectiveness of newer influenza vaccines have been published. One presented evidence from nine studies that adjuvanted vaccines were more effective than standard‐dose vaccines in reducing influenza‐related outcomes [6]. Another showed mixed evidence on the relative effectiveness of cell‐based vaccines versus standard‐dose egg‐based vaccines, but the relative effectiveness (rVE) of QIVc versus QIVe was around 8.5% in meta‐analyses [7]. Results on the effectiveness of QIVr varied in a recent review; a real‐world study found no difference in rVE of QIVr versus QIVe, while a randomised controlled trial found increased rVE with QIVr [8].

For the 2022/2023 and 2023/2024 seasons aQIV or QIVr vaccines were recommended first line for those aged 65+ years, with QIVc second‐line. QIVe was specifically advised against for adults aged 65+ years. QIVc or QIVr vaccines were recommended for adults aged 18–64 years (QIVe second‐line), and LAIV for children aged 2–17 years (or QIVc where LAIV was contraindicated or unsuitable; QIVe third‐line) [2].

While waning has been examined previously in the UK [9, 10], the relatively early, A(H3N2)‐dominated, 2022/2023 season in England, and the long season of 2023/2024, each provide distinctive circumstances for evaluation of in‐season waning. Additionally, discontinuation of the influenza vaccine offer to those aged 50–64 years in 2023/2024 provides an opportunity to evaluate the effectiveness of past season vaccination in healthy adults. Notably the A(H3N2) and B components of the 2023/2024 northern hemisphere vaccine remained the same as for the 2022/2023 vaccine, while the A(H1N1) component was updated [11].

Both VE and serological studies have found evidence for in‐season waning. A meta‐analysis found that VE was higher 15–90 days post vaccination than at 91–180 days post vaccination [12]. A large Canadian study over multiple seasons similarly found declines in VE in adults within season [13]. Further, a meta‐analysis of serological studies provided evidence of incremental reductions in seroprotection after 180 and 360 days post vaccination compared with days 21–42 [14]. Hence protection in vulnerable adults may be optimised by delivering vaccines closer to the time of influenza circulation [12].

This paper presents VE against hospitalisation in England, across two seasons: 2022/2023 and 2023/2024. Here, we evaluate influenza VE by vaccine type, by time since vaccination, and by combination of past and current season vaccination during both the 2022/2023 and 2023/2024 seasons. VE analyses are presented covering ages 2–17, 18–64 and 65+ years, for all influenzas, influenza A and B and influenza A subtypes. We also explore longer term waning in adults aged 50–64 years in 2023/2024.

## 2 Methods

### 2.1 Study Design and Target Population

A test‐negative design (TND) case–control study was used to estimate VE, with methods resembling those used for the 2021/2022 influenza season [15].

The study population was defined as residents in England ≥ 2 years of age (on 31 August 2022 and 2023) who had a respiratory swab taken, during the main periods when influenza was circulating in each season (between Week 402,022 and Week 152,023 or Week 402,023 and Week 172,024) which was tested for influenza using nucleic acid amplification testing, and had a record of hospitalisation up to 14 days after the swab or up to 2 days before a swab. A case was defined as a person with laboratory confirmed influenza infection and a control was defined as a person who was tested for influenza infection and was negative for influenza infection.

### 2.2 Data Sources

Our national‐level healthcare datasets comprised laboratory testing data, vaccine registry and hospitalisation data linked using NHS number and date of birth. Two sources of laboratory testing data were used to identify influenza‐positive cases and influenza‐negative controls. First, the Respiratory DataMart (RDM), a sentinel laboratory surveillance system, with 17 participating laboratories [16], and second, the Second‐Generation Surveillance System (SGSS), which records laboratory outcomes across England [17]. Note that results in RDM are largely (although not entirely) a subset of those in SGSS, with additional influenza A subtype information available for some labs. Secondary Uses Service (SUS) [18], a data repository for secondary care in England, was used to identify hospitalisations with a discharge code (ICD‐10) compatible with acute respiratory infection (see Table S2), within 14 days of a respiratory swab, or where a swab was taken up to 2 days after admission. The Immunisation Information System (IIS), a national individual level vaccine register, provided vaccination histories for seasons 2021/2022 through 2023/2024, clinical risk status and demographic variables [19]. Additional information on the data sources is presented in Table S1.

### 2.3 Exclusions

Records were excluded if no test date was given, or if influenza status was not fully known (i.e., controls required both influenza A and B negative results). Testing data were first deduplicated such that no more than one test per person per 28‐day period was retained, and a new positive test was kept over a new negative test so that any new positive test around the time of a respiratory hospital admission defined a case. We then further restricted the inclusion of the first of each influenza A(H1N1), A(H3N2) and B positive test, and where influenza A was not subtyped, we ensured positive tests were at least 6 weeks apart. We further excluded tests with no linkage to IIS, within 0–13 days of vaccination, plus adults with a record of receiving LAIV and children that received a recombinant or adjuvanted vaccine. Given the association between COVID‐19 and influenza vaccination and its impact on the vaccination status of noncases, SARS‐CoV‐2 positive controls were removed.

### 2.4 Statistical Analysis

Cases and controls were described by key covariables with differences between groups tested using chi‐squared tests. The number of influenza‐positive cases and negative controls were plotted by week.

Logistic regression was used to calculate the odds ratio for vaccination in cases compared with controls. This was used to calculate VE as VE = 1 − OR, with a 95% confidence interval. Our estimates were adjusted for week of sample, age group (2–3, 4–6, 7–10, 11–15, 16–17, 18–34, 35–49, 50–64, 65–74, 75–84 and 85+ years), region and clinical risk status (classified as none, in a risk group or immunosuppressed according to definitions outlined for vaccine eligibility [20]). Additional variables (sex, ethnicity and deprivation) were considered for adjustment, but they did not change the vaccine effect by more than 1%, so their inclusion was not deemed necessary. Only complete records were retained. Results are only reported where the expected number of case/control vaccinated/unvaccinated cell counts were all > 8.

Separate analyses were carried out for children aged 2–17, adults aged 18–64 and adults aged 65+, years combining SGSS and RDM testing data and for RDM only. We estimated an overall VE (any vaccine type), VE by vaccine type, by influenza type and subtype. To explore waning VE, we estimated influenza type/subtype specific VE by time since vaccination, categorised as 2–8, 9–12, 13–16 and 17–33 weeks; these groups were chosen to divide events into four groups of roughly equal size over the two seasons. We also give relative VE (rVE) using as the reference category the first postvaccination period (2–8 weeks post vaccination); we consider rVE with 95% CI, entirely below 0 as evidence for waning. VE by combinations of past and current season vaccination were also estimated. In 50–64 year olds during 2023/2024, we also considered extended VE by time since vaccination over two seasons, grouping by 2–12, 13–31, 32–52 and 53–84 weeks post vaccination; 2023/2024 season vaccinations were almost all within 31 weeks.

We combined results on VE by vaccine type over the two seasons using a one‐stage stratified intercepts meta‐analysis [21]. We also give a rVE for vaccine types, using as the reference category the vaccine given most commonly within an age group (LAIV for children, QIVc in adults 18–64 and aQIV in adults 65+). We regard rVE with 95% CI, entirely above or below 0 as evidence for a difference in VE by vaccine type.

### 2.5 Sensitivity Analyses

Several additional sensitivity analyses were run to further explore waning within each season. First, we additionally used the self‐controlled case series (SCCS) design to explore relative waning; methods and results are given in Box S1. Second, using the TND, time since vaccination was divided into two periods: 2–12 and 13–33 weeks, which aligns with past studies [12]. Third, following methods outlined by Ray et al. [22], we used a vaccinees‐only TND conditional on the date of test, including only those vaccinated before influenza activity began to rise, which ensured similar susceptibility of all individuals included regardless of the timing of vaccination [22, 23, 24]. Finally, a sensitivity analysis was carried out amending the first postvaccination period to 3–8 weeks in children, to ensure elimination of any vaccine virus detected following LAIV receipt.

### 2.6 Software

Microsoft SQL Server Management Studio 18 was used for data linkage. STATA Version 14.2/18 was used for TND data analyses, and R Version 3.6.3 for SCCS analyses (package SCCS) and forest plots (package forestploter).

## 3 Results

### 3.1 Exclusions and Descriptive Statistics

Exclusions of cases and controls are listed in Tables S3 and S4. Overall, 100,581 and 113,494 samples were available after exclusions for the 2022/2023 and 2023/2024 seasons, respectively. Descriptive statistics of cases (by influenza type) and controls included are shown in Tables S5–S10, with separate tables for each age cohort and season. In general, during the 2022/2023 season, influenza positivity was highest during the month of December, among secondary school‐aged children aged 11–17 or young adults aged 18–34 and among the unvaccinated. During the 2023/2024 season, influenza positivity was lower than for 2022/2023 but was highest in young adults and among the unvaccinated. There was little difference in influenza positivity by sex or deprivation status.

**FIGURE 1:**
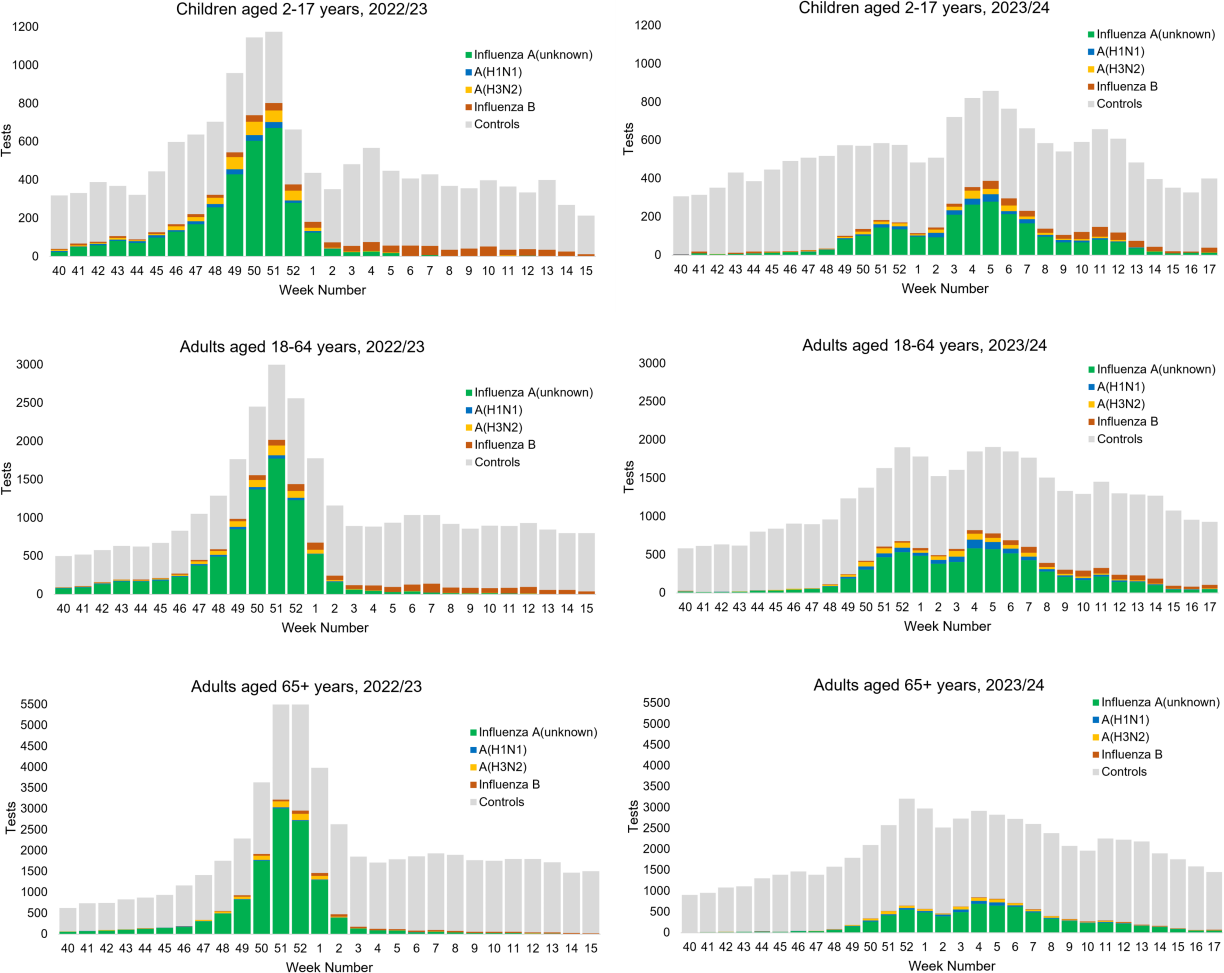
Number of influenza detections and controls, by age and season.

Case and control counts by week included in the analyses for the 2022/2023 and 2023/2024 seasons are shown in Figure 1, while Figure S1 illustrates the relative circulation of A(H1N1) and A(H3N2). In the 2022/2023 season, influenza A(H3N2) was prevalent during September 2022 through to January 2023, with some A(H1N1) cocirculation; influenza activity peaked sharply in late December 2022 [25]. During February–April 2023 influenza activity was lower, and influenza B was predominant. In contrast, the 2023/2024 season's influenza activity did not increase as early and was more sustained during the late season, without a sharp peak. Influenza A was predominant, with cocirculation of influenza A(H1N1) and A(H3N2); activity was highest from late December 2023 through January 2024. Influenza B circulated at a low level, increasing from February 2024 onward. During both seasons, there were relatively few influenza B detections among those aged 65+ years.

Figure S2 illustrates the timing of vaccination in each age cohort. Those aged 65+ years tended to be vaccinated early in the season, primarily during September to October. Most child vaccinations were administered during October to November, but during the 2022/2023 season delivery continued into January 2023. There were no major differences in the time since vaccination by vaccine type for any of the age cohorts in either the 2023/2023 or 2023/2024 seasons (Figure S3). Age and risk group by vaccine type for each cohort and season are shown in Table S11.

### 3.2 VE by Influenza Type

**FIGURE 2:**
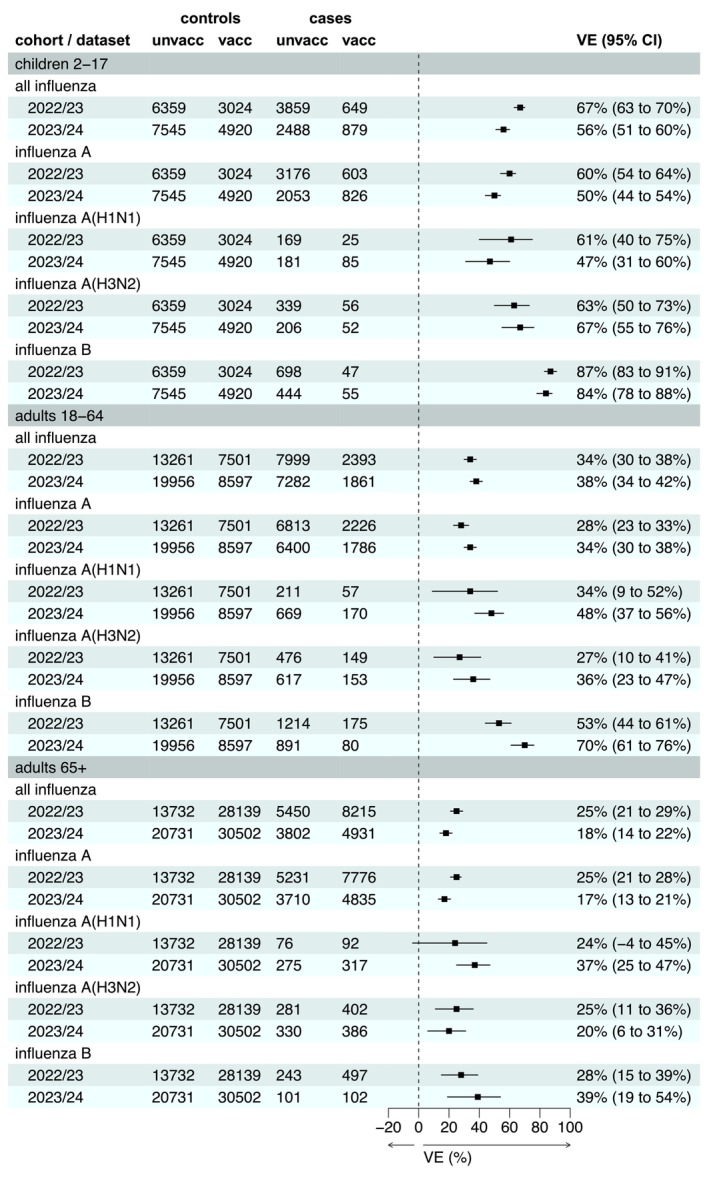
Vaccine effectiveness by age group, for all influenzas and by influenza type/subtype: influenza A (all subtypes), A(H1N1), A(H3N2) and influenza B. VE = vaccine effectiveness adjusted for week, age group, region and clinical risk status.

The VE estimates overall, by influenza type and by influenza A subtype across the whole of each season are shown in Figure 2. The overall VE for children aged 2–17 years was 67% (95% CI, 63%–70%) in 2022/2023 and 56% (95% CI, 51%–760%) in 2023/2024. The overall VE for adults aged 18–64 was 34% (95% CI, 30%–38%) in 2022/2023 or 38% (95% CI, 34%–42%) in 2023/2024. The overall VE for adults aged 65+ years was positive, but lower at 25% (95% CI, 21%–29%) in 2022/2023 and 18% (95% CI, 14%–22%) in 2023/2024. VE estimates for influenza A were consistently lower than those for influenza B. Within each age group, VE estimates were broadly similar for influenza A(H1N1) and A(H3N2) in 2022/2023, whereas in 2023/2024 A(H1N1), VE point estimates were slightly higher in adults while lower in children. However, influenza A subtypes are frequently unknown, leading to more uncertain VE estimates with wide confidence intervals.

Overall VE analyses were repeated using RDM data alone; results are shown in Figure S4. VE estimates were similar to the combined data among children and working‐age adults. VE point estimates among those age 65 years and older were higher using the RDM data alone, with an overall VE of 33% (24%–40%) in 2022/2023 and 29% (22%–36%) in 2023/2024, though confidence intervals overlap with those using the combined SGSS and RDM data.

### 3.3 VE by Vaccine Type

**FIGURE 3:**
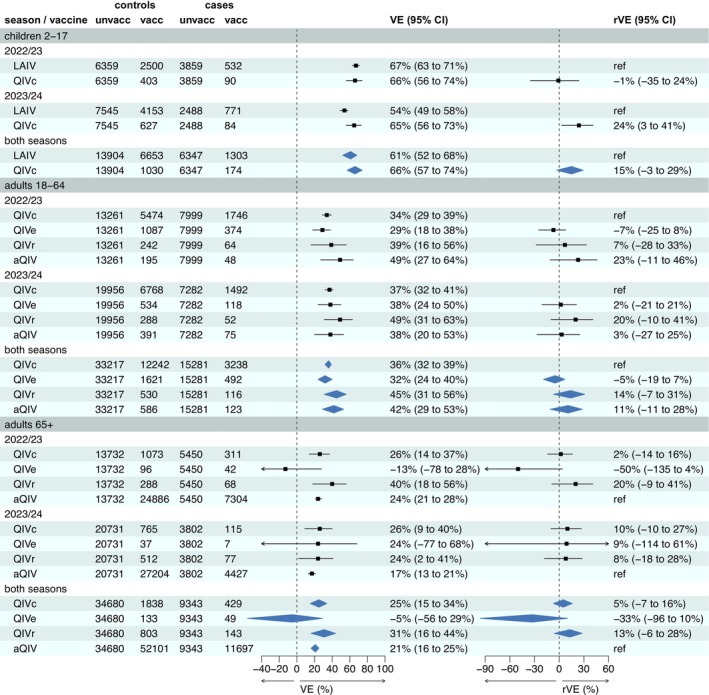
Vaccine effectiveness by broad age group, vaccine type and season, including meta‐analysis of two seasons. VE = vaccine effectiveness adjusted for week, age group, region and clinical risk status. aQIV = adjuvanted influenza vaccine; LAIV = live attenuated influenza vaccine; QIVc = cell‐based influenza vaccine; QIVe = standard‐dose egg‐based influenza vaccine; QIVr = recombinant influenza vaccine; rVE = relative vaccine effectiveness.

VE by vaccine type is given in Figure 3, reported as both VE versus unvaccinated, and rVE, and including a meta‐analysis for both seasons. Among children aged 2–17, VE for LAIV and QIVc did not differ (rVE QIVc vs. LAIV −1% [95% CI, −35% to 24%]), while VE for QIVc was higher during the 2023/2024 season (rVE QIVc vs. LAIV 24% [95% CI, 3%–41%]). Among adults aged 18–64, VE did not differ for those receiving QIVc, QIVe, QIVr and aQIV for both seasons; in our meta‐analysis, rVE versus QIVc was −5% (95% CI, −19% to 7%) for QIVe, 14% (95% CI, −7% to 31%) for QIVr and 11% (95% CI, −11% to 28%) for aQIV. Note that of those aged 18–64 that received aQIV, most were towards the upper end of the age bracket (Table S11). Among individuals aged 65+ years, aQIV, QIVc and QIVr VE did not differ; in our meta‐analysis, rVE of QIVc versus aQIV was 5% (95% CI, −7% to 16%) and rVE of QIVr versus aQIV was 13% (95% CI, −6% to 28%). rVE of QIVe versus aQIV for those aged 65+ years appeared lower at −33% (95% CI, −96% to 10%); however, a small number received this vaccine in this cohort, and there was no significant difference. Overall, there was no significant difference in rVE between vaccine types in any age cohort after combining estimates for both seasons.

### 3.4 VE Over Time

**FIGURE 4:**
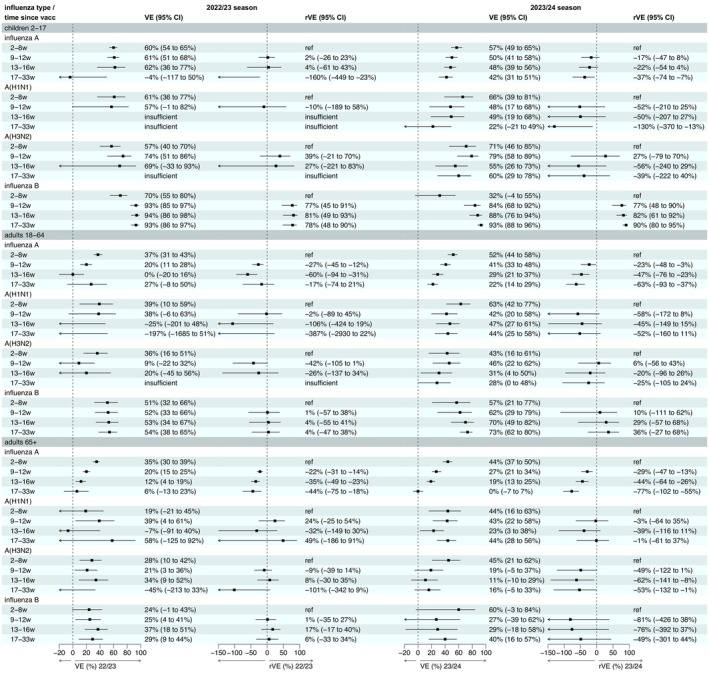
Vaccine effectiveness by time since vaccination, SGSS data. VE = vaccine effectiveness adjusted for week, age group, region and clinical risk status.

VE by time since vaccination is given in Figure 4, case and control counts by vaccination status can be found in Figures S5 and S6. There was some evidence of a reduction in effectiveness by time since vaccination against influenza A in both seasons, most notably in adults, but no evidence of a reduction in VE against influenza B in any age group. In children, VE against influenza B was lowest during the period 2–8 weeks post vaccination; this increased in the sensitivity analysis where Week 2 was removed, as shown in Figure S7, but VE still remained lower than in the later periods. In the same sensitivity analysis, VE against influenza A did not change by more than 2%. VE by the periods 2–12 weeks post vaccination and 13 or more weeks post vaccination is shown in Figure S8. Analyses of A(H1N1) and A(H3N2) VE by time since vaccination had small case numbers in some categories leading to uncertain estimates. The SCCS analysis yielded broadly similar findings of reductions in influenza A VE by time since vaccination in adults (Figures S5 and S6), though the reduction in influenza A VE by time since vaccination was a little less steep than the TND. The vaccinees‐only TND sensitivity analysis (Figures S9 and S10) did not find any evidence of waning in children, but results help confirm the presence of decreasing influenza A VE by time since vaccination in adults.

In the 2022/2023 season, while there is evidence that past season vaccination offers limited protection, there is also clear evidence that current and past season vaccination together provide protection beyond past season vaccination alone (Figure S11). For example, influenza A VE for past season vaccination alone versus unvaccinated both seasons was 42% (95% CI, 33%–49%) in ages 2–17, 17% (95% CI, 10%–25%) in ages 18–64 and 23% (95% CI, 16%–28%) in ages 65+ years, and rVE for current season vaccination versus past season vaccination alone was 50% (95% CI, 39%–58%) in ages 2–17, 19% (95% CI, 10%–27%) in ages 18–64 and 15% (95% CI, 9%–20%) in ages 65+. Again, in 2023/2024, past season vaccination appears to offer protection against both influenza A and B in all age groups, and among children, current and past season vaccination is more effective than past season vaccination alone. For example, influenza A VE for past season vaccination alone versus unvaccinated both seasons was 40% (95% CI, 29%–48%) in ages 2–17, 35% (95% CI, 29%–40%) in ages 18–64 and 44% (95% CI, 39%–48%) in ages 65+ years, and rVE for current season vaccination versus past season vaccination alone was 45% (95% CI, 34%–54%) in ages 2–17. However, the 2023/2024 season differs among adults: There is no evidence that current season vaccination is more effective than past season vaccination alone (except for A(H1N1) in elderly adults), and among those aged 65+, VE for combined current and past season vaccination is lower than VE for past season vaccination alone. For influenza A, rVE for current season vaccination versus past season vaccination alone was 5% (95% CI, −5% to 14%) for ages 18–64 and −9% (95% CI, −16% to −2%) for ages 65+ years.

**FIGURE 5:**
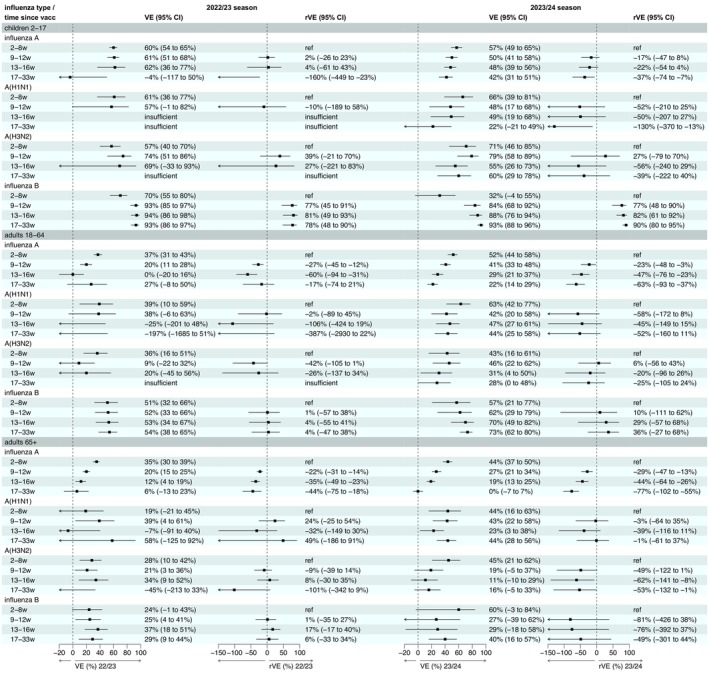
Vaccine effectiveness in individuals aged 50–64 years during the 2023/2024 season, by time since vaccination taking both 2022/2023 and 2023/2024 season vaccination, SGSS data.

Figure 5 shows VE for the 2023/2024 season among those aged 50–64 by time since last vaccination up to 84 weeks post vaccination. This was a population that were offered influenza vaccination during the COVID‐19 pandemic for three seasons from 2020/2021 to 2022/2023, but this was discontinued in 2023/2024. We note that current season vaccination is typically within 31 weeks and past season vaccination extends up to 84 weeks; the A(H3N2) and B vaccine components remained the same while the A(H1N1) component was updated for 2023/2024. Effectiveness against influenza A was highest within 2–12 weeks of (current season) vaccination; however, there was little difference in VE from 13–31 weeks post vaccination to 53–84 weeks post vaccination. Case numbers were low for influenza B and VE was imprecise.

## 4 Discussion

Key findings from our analysis are as follows: Firstly, influenza vaccines offered protection against influenza hospitalisations in all age groups. Overall VE over each season was highest in children and lowest in older adults aged 65+ years. Secondly, there was no clear evidence of a difference in VE between the different enhanced influenza vaccines that have been used in adults in England (QIVc, aQIV and QIVr) during the 2022/2023 and 2023/2024 seasons. Thirdly, notable in‐season waning in effectiveness against influenza A was seen in adults (with much higher effectiveness in the first few weeks after vaccination), though there was some evidence of limited residual protection of previous seasons' vaccine; and there was no evidence of waning in influenza B VE.

Effectiveness of influenza vaccination in preventing influenza‐associated hospitalisation in children was good; consistent with previous UK‐based studies [15, 26, 27] and a European network where VE in children was 69% (95% CI, 60–76) in 2022/2023 and 70% (95% CI, 61–78) in 2023/2024 [28, 29]. Protection was highest against influenza B, which is in line with other studies [28, 29, 30]. In our study, effectiveness was higher for children in the 2022/2023 season than in the 2023/2024 season. There was some difference in the epidemiology of circulating influenza strains in England between the seasons; influenza A was dominant in both, with the A(H3N2) subtype dominating in 2022/2023 while there was an equal split of A(H1N1) and A(H3N2) in 2023/2024 [31, 32]. This may partly explain the slight differences in VE as low effectiveness of LAIV against A(H1N1)pdm09 has been seen previously [33]. However, VE against A(H1N1) within a European network in children during the 2023/2024 season was 73% (95% CI, 61%–82%), higher than in our study. Further, it is likely that prior immunity and vaccination history are important for overall influenza immunity [34]. This may partly explain why VE is higher in children, who have limited past exposures compared with older adults. Reduced influenza circulation from spring 2020 to summer 2022 may have led to an atypically high proportion of infection‐naive young children at the beginning of the 2022/2023 season. The importance of vaccine history was also seen in children, where those vaccinated two seasons in a row had higher VE against influenza A than children who had received only one vaccine in the last two seasons. LAIV VE has previously been found to be higher in children vaccinated in both the current and prior season compared with those vaccinated only in the current season [35].

We found evidence of protection against influenza hospitalisation in adults, including against influenza A and B, although protection was much lower than for children, and appreciably lower for older adults aged 65+ years (overall and by influenza type) compared with younger adults aged 18–64 years. While findings of lower VE in adults are typical, our adult all influenza VE estimates were generally lower than those seen in Europe, Canada or the United States [28, 29, 30, 36, 37, 38]. The A(H1N1) vaccine strain was updated from A/Victoria/2570/2019(H1N1)pdm09‐like in 2022/2023 to A/Victoria/4897/2022 (H1N1)pdm09‐like for 2023/2024 [11]. Our point estimates for VE against A(H1N1) in adults were slightly higher in 2023/2024 than in 2022/2023; this agrees with results for adults aged 65+ years in Europe but conflicts with their results for adults aged 15–64 where VE against A(H1N1) was higher for the 2022/2023 season [28, 29]. Our influenza A(H3N2) VE estimates in adults were within 5% of A(H3N2) VE estimates for Europe [28, 29]. Our influenza A subtype results are limited by the wide confidence intervals around our estimates due to the small numbers of tests with subtyping data available.

Notably, we did not observe statistically significant differences in VE by vaccine type for children, working‐age adults or adults aged 65+ years in a meta‐analysis over two seasons. Our study provides reassuring evidence that all recommended vaccines provide some protection against severe influenza. Standard‐dose egg‐based vaccines have long been associated with low effectiveness, especially in older adults, and a number of enhanced influenza vaccines have been developed for use including high‐dose, adjuvanted and recombinant vaccines. However, there are limited data on the real‐world relative effectiveness—a systematic review was conducted in April 2024, which found no data on head‐to‐head comparisons between the different new and/or enhanced vaccines [39]. Three studies on influenza VE in adults aged 65+ years from the United States, conducted between 2017 and 2020, found overall similar VE for aQIV and high‐dose vaccines with some evidence of higher VE for QIVr but with less certain results [40, 41, 42]. The head‐to‐head comparisons in our study are limited by the fact that in each age cohort, one vaccine type was very dominant (LAIV for children, QIVc for working‐age adults and aQIV for older adults). Even so, enough doses of different vaccine types were given to produce reasonably precise and comparable estimates.

There was evidence of waning against influenza A in adults. Notably, after 17–33 weeks from vaccination, VE for influenza A in elderly adults was close to 0% in both seasons, indicating that individuals who received a vaccine in the current season had a comparable level of protection against influenza with those who were not vaccinated in the current season. VE in the first 2 months after vaccination was substantially higher (35%–45%) than the overall estimates for each season. This suggests vaccination closer to influenza transmission periods would optimise protection: For example, if the vaccine is delivered during Week 40 (which is typical in this age group), our analyses suggest that protection against influenza A from the current season's dose may not persist beyond the end of January.

Despite the evidence on waning, we saw evidence that vaccination in the previous season continued to provide limited protection against influenza A in the latest season versus those with no record of vaccination in either season. These results suggest that influenza vaccines have the potential to induce long‐term protection up to one and a half years, depending upon how well a previous season's vaccine matches this season's circulating strains. We have previously found serological evidence that influenza antibodies from past season vaccination can persist although at lower levels on average than in those with current season vaccination [15]. Where long‐term protection exists, our analyses of in‐season waning are likely measuring a short‐term boost that wanes back to some background level of residual protection from past vaccination. However, mixed results on the protection of only prior season vaccination have previously been seen across six seasons in the United States [43], suggesting that favourable circumstances for past season vaccination effectiveness vary.

The population of healthy adults aged 50–64 who were extended an offer of influenza vaccination from 2020/2021 through 2022/2023 seasons only in the United Kingdom, provided us with a unique opportunity to explore longer term protection and waning in the 2023/2024 season. Findings agreed with those on combinations of current and past season vaccination in that residual long‐term protection was found against influenza A.

We found no evidence of in‐season waning against influenza B. VE against influenza B was lowest during the period 2–8 weeks post vaccination in children. We suspected that some influenza positives were due to shedding of LAIV vaccine virus beyond 13 days post vaccination, so we removed Week 2 (14–20 days post vaccination) in sensitivity analyses. Viral shedding of LAIV vaccine strains beyond 13 days post vaccination has previously been observed, although it was uncommon [44]. This sensitivity analysis found increased influenza B VE during Weeks 3–8 post vaccination but VE still remained lower than in later periods; there was little impact on findings for influenza A.

## 5 Strengths and Weaknesses

The main strength of this study is the use of national‐level healthcare data. This has enabled us to estimate the real‐world effectiveness of influenza vaccines across two seasons for the population of England and the size of the study allows us to estimate differences by vaccine type, influenza subtype and timing after vaccination. The main limitations relate to this being an observational study. There may be some biases that we cannot entirely control or adjust for.

The IIS is unlikely to cover vaccinations privately offered within workplaces or abroad unless these data were fed back to the GP practice. Private vaccination of children and adults age 65+ years is rare in the United Kingdom, and working‐age adults with risk factors for severe influenza are eligible for free vaccination, so any effect on estimates is likely to be modest.

RDM includes sentinel RT‐PCR testing for types A and B; some laboratories further subtype A(H1N1) and A(H3N2). Early in the 2022/2023 season, there were known issues with A(H1N1) detection using certain equipment; no DataMart laboratories were impacted. While SGSS has the advantage of size, influenza subtypes are not frequently reported. Influenza positivity was higher in SGSS than in RDM; while reporting guidelines state that all influenza‐negative tests should be reported, it is possible that not all negatives are reported by laboratories. Despite the limitations of the SGSS data, its size is useful for subgroup analyses, such as for exploring rVE between vaccines.

The TND ideally requires tests from patients presenting with similar symptoms. Despite using acute respiratory coded hospitalisations, we do not know whether influenza‐like illness was the main underlying reason for hospitalisation. Timing of symptom onset is unknown, which may affect the sensitivity of tests.

Analyses of waning may be affected by any temporal trends or drift in the specific viruses circulating over the course of a season, which will complicate interpretation [45]. Figure S1 aids understanding of these temporal trends for influenza A; during the 2022/2023 season, there were proportionately more A(H1N1) towards the end of the season whereas during 2023/2024, the proportions of A(H1N1) and A(H3N2) remained similar throughout. Further, those vaccinated early and late during the season will not have contributed equal case numbers to the 2–8 weeks and 15 or more weeks post vaccination periods, especially during the sharp 2022/2023 season, and factors influencing timing of vaccination may not have been fully taken into account. Influenza A(H3N2) during the 2023/2024 season provides good circumstances to explore waning: circulating viruses almost all belonged to the same clade and moderate circulation persisted over a 4‐month period, while there were multiple A(H3N2) clades in circulation during 2022/2023 [31, 32]. However, data on influenza A subtype were often lacking, and genetic characterisation of positive influenza tests in our study was not available. Depletion of the susceptible population as those infected gain natural immunity over the study period will create bias that exaggerates waning using both the TND [24] and SCCS designs (in SCCS, this is similar to the bias induced by including first events only [46]). However, the impact of any depletion‐of‐susceptibles bias is likely to be small relative to the observed effect size of influenza A waning [22, 24, 46]. The sensitivity analysis including only individuals vaccinated before influenza circulation increased, which should not be biased by depletion of the susceptible population throughout the season, did not alter conclusions for adults.

Although the specificity of tests used is likely to be very high, small numbers of false positive tests will bias VE towards the null when incidence rates are very low [15]. Our analyses for influenza type/subtype may be impacted by these biases during periods of low circulation for that particular type/subtype, and this may partly explain the low VE for influenza B 2–8 weeks post vaccination because most vaccinations were given early in the season when influenza B circulation was low.

In summary, our study shows that influenza vaccines continue to offer protection against hospitalisation in all age groups, with little evidence that any of the enhanced vaccines in use in England outperform the others. The level of protection in adults was relatively low, though it was much higher in the first 2 months after vaccination. This strengthens the argument for initiating the delivery of influenza vaccines closer to the expected peak of influenza activity to ensure that optimal protection is provided when the risk of influenza is greatest. Our results also support the development of vaccines with higher effectiveness and longer duration of protection.

## Author Contributions

**Heather J. Whitaker:** conceptualization, methodology, formal analysis, supervision, visualization, writing – original draft, writing – review and editing. **Freja C. M. Kirsebom:** data curation, validation, formal analysis, writing – original draft, writing – review and editing. **Katie Hassell:** data curation, methodology, validation. **Catherine Quinot:** data curation, validation. **Julia Stowe:** data curation, supervision, validation, methodology. **Katja Hoschler:** investigation, validation, data curation. **Maria Zambon:** data curation, supervision, investigation, validation, writing – review and editing. **Nick J. Andrews:** conceptualization, methodology, supervision, writing – review and editing. **Conall H. Watson:** conceptualization, supervision, writing – review and editing. **Jamie Lopez Bernal:** conceptualization, supervision, writing – review and editing.

## Ethics Statement

This work was an evaluation of a vaccination programme. The UK Health Security Agency holds permissions under section 251 of the 2006 NHS Act, and under Regulation 3 of the Health Service (Control of Patient Information) Regulations 2002 (www.legislation.gov.uk/uksi/2002/1438/regulation/3/made), to process patient information for such purposes.

## Conflicts of Interest

The Immunisations and Vaccine‐Preventable Diseases division at UKHSA has received cost‐recovery payment from CSL Seqirus for analysis undertaken for regulatory review. No other authors had conflicts to declare.

## Supporting information


**Table S1:** Data sources.
**Table S2:** ICD‐10 code list for inclusion in the study.
**Table S3:** Exclusions for 2022–2023 data (SGSS and DataMart).
**Table S4:** Exclusions for 2023–2024 data (SGSS and DataMart).
**Table S5:** Descriptive statistics for ages 2–17, 2022/2023 season.
**Table S6:** Descriptive statistics for ages 18–64, 2022/2023 season.
**Table S7:** Descriptive statistics for ages 65+, 2022/2023 season.
**Table S8:** Descriptive statistics for ages 2–17, 2023/2024 season.
**Table S9:** Descriptive statistics for ages 18–64, 2023/2024 season.
**Table S10:** Descriptive statistics for ages 65+, 2023/2024 season.
**Figure S1:** Percentage of influenza A(H1N1) or A(H3N2) detections by month, using data from four RDM laboratories that consistently subtype influenza A (includes nonhospitalised patients).
**Figure S2:** Percentage of vaccinations given by week and age group among vaccinated noncases (includes nonhospitalised noncases), overlaid on a count of hospitalised influenza‐positive cases.
**Figure S3:** Time between vaccination and test (weeks) by vaccine type for each age cohort and season, all vaccinated cases and controls.
**Table S11:** Descriptive table of age group and risk status by influenza vaccine type, for each age cohort and season. Count (*n*) with column percentage (%).
**Figure S4:** Vaccine effectiveness by broad age group, for all influenzas and by influenza type/subtype: influenza A (all subtypes), A(H1N1), A(H3N2) and influenza B, based on DataMart data alone.
**Box S1:** Additional information and methods on self‐controlled case series analyses for waning relative VE.
**Figure S5:** Waning vaccine effectiveness: comparison of test negative and SCCS, 2022/2023 season.
**Figure S6:** Waning vaccine effectiveness: comparison of test negative and SCCS, 2023/2024 season.
**Figure S7:** Waning vaccine effectiveness: original analysis (upper) and sensitivity analysis (lower) in children aged 2–17 that excludes Week 2 post vaccination (the reference category for rVE is 3–8 weeks).
**Figure S8:** Waning vaccine effectiveness: two categories.
**Figure S9:** Waning vaccine effectiveness 2022/2023 season: original analysis and sensitivity analysis including only those vaccinated by 7 November 2022 (when influenza activity began to rise).
**Figure S10:** Waning vaccine effectiveness 2023/2024 season: original analysis and sensitivity analysis including only those vaccinated by 4 December 2023 (when influenza activity began to rise).
**Figure S11:** Combined current and past season vaccine effectiveness.

## Data Availability

Data supporting this study cannot be made available due to ethical and legal reasons.
