# Supplementary material for: Effectiveness of Influenza Vaccines and Duration of Protection Against Hospitalisation in England: 2022/2023 and 2023/2024 Seasons
Source: Influenza Other Respir Viruses. 2025 Nov 27;19(12):e70194. doi: 10.1111/irv.70194 (PMC12658907; doi:10.1111/irv.70194)
Supplement: Supplementary file 1 — Table S1: Data sources. Table S2: ICD‐10 code list for inclusion in the study. Table S3: Exclusions for 2022–2023 data (SGSS and DataMart). Table S4: Exclusions for 2023–2024 data (SGSS and DataMart). Table S5: Descriptive statistics for ages 2–17, 2022/2023 season. Table S6: Descriptive statistics for ages 18–64, 2022/2023 season. Table S7: Descriptive statistics for ages 65+, 2022/2023 season. Table S8: Descriptive statistics for ages 2–17, 2023/2024 season. Table S9: Descriptive statistics for ages 18–64, 2023/2024 season. Table S10: Descriptive statistics for ages 65+, 2023/2024 season. Figure S1: Percentage of influenza A(H1N1) or A(H3N2) detections by month, using data from four RDM laboratories that consistently subtype influenza A (includes nonhospitalised patients). Figure S2: Percentage of vaccinations given by week and age group among vaccinated noncases (includes nonhospitalised noncases), overlaid on a count of hospitalised influenza‐positive cases. Figure S3: Time between vaccination and test (weeks) by vaccine type for each age cohort and season, all vaccinated cases and controls. Table S11: Descriptive table of age group and risk status by influenza vaccine type, for each age cohort and season. Count (n) with column percentage (%). Figure S4: Vaccine effectiveness by broad age group, for all influenzas and by influenza type/subtype: influenza A (all subtypes), A(H1N1), A(H3N2) and influenza B, based on DataMart data alone. Box S1: Additional information and methods on self‐controlled case series analyses for waning relative VE. Figure S5: Waning vaccine effectiveness: comparison of test negative and SCCS, 2022/2023 season. Figure S6: Waning vaccine effectiveness: comparison of test negative and SCCS, 2023/2024 season. Figure S7: Waning vaccine effectiveness: original analysis (upper) and sensitivity analysis (lower) in children aged 2–17 that excludes Week 2 post vaccination (the reference category for rVE is 3–8 weeks). Figure S8: Waning vaccine eff [file IRV-19-e70194-s001.docx]

**Supplementary material: Effectiveness of influenza vaccines and duration of protection against hospitalisation in England: 2022/23 and 2023/24 seasons**

***Data***

**Table S1. Data sources**

| Data Source | Description | Variables |
| --- | --- | --- |
| SGSS | Second Generation Surveillance system (SGSS) is the national laboratory reporting system used in England to capture routine laboratory data on infectious diseases, including influenza. More most pathogens, SGSS only reports positive test results but for influenza and COVID-19 laboratories report positive and negative test results. | NHS number, date of birth (age), date of test, PCR test result, influenza sub-typing information, information on previous positivity, geographic region. |
| Respiratory Datamart | The Respiratory DataMart sentinel system was initially set up in 2009 to automate the collection of all influenza A(H1N1)pdm09 laboratory testing information in England. It is now an important sentinel laboratory surveillance tool, monitoring all major respiratory viruses in England, with 17 laboratories contributing data at present through weekly automatic electronic outputs. This includes the national reference laboratory (Respiratory Virus Unit (RVU), UKHSA Colindale), regional public health laboratories based on hospital sites, and NHS partner laboratories. | NHS number, date of birth (age), date of test, PCR test result, influenza sub-typing information, information on previous positivity, COVID-19 test result data, geographic region. |
| IIS vaccination record | The Immunization Information System (IIS) contains demographic information on the whole population of England who are registered with a general practice physician in England and is used to record all influenza and COVID-19 vaccinations. | Vaccination history including the data administered, manufacturer and vaccine type. Demographic data including index of multiple deprivation, ethnicity, geographic region, date of birth, clinical risk group status. Clinical risk groups for influenza include a range of conditions as described in the Green Book. |
| Secondary Care Hospital Admission Data (SUS) | SUS is the national electronic database of hospital admissions that provides timely updates of ICD-10 codes for completed hospital stays for all NHS hospitals in England. Up to 24 ICD-10 diagnoses fields can be completed in SUS for each admission with the first diagnosis field indicating the primary reason for admission. Where multiple admissions linked to the same sample date the first admission after the sample date was retained. Data were restricted to those with ICD-10 codes from table S1.2 in any diagnosis field. | Admission date and ICD-10 diagnosis data. |

**Table S2, ICD-10 code list for inclusion in the study**

| **ICD-10 Codes** | **ICD-10 code description** |
| --- | --- |
| B34 | Viral infection, unspecified site |
| A419 | Sepsis |
| R572 | Septic shock |
| J00-J06 | *Acute upper respiratory infections* |
| J09-J18 | *Influenza and pneumonia* |
| J20-J22 | *Other acute lower respiratory infections* |
| J80 | *Adult respiratory distress syndrome* |
| J960, J969 | Respiratory failure |

***Exclusions***

**Table S3** Exclusions for 2022-2023 data (SGSS and Datamart)

|  | n excluded | N in sample |
| --- | --- | --- |
| to start |  | 578,681 |
| ages 0 to 1 or no age | 1,257 | 577,424 |
| sample date before study start | 0 | 577,424 |
| sample date after study end | 0 | 577,424 |
| influenza status not fully known | 8,559 | 568,865 |
| de-duplication to 1 sample per person per day | 83,634 | 485,231 |
| remove control samples within 7 days of another sample | 77,359 | 407,872 |
| de-duplication to 1 control sample per person per 28-day period | 31715 | 376,157 |
| de-duplication to 1 case sample per person per 28-day period | 4,014 | 372,143 |
| remove additional controls up to 28 days after a positive test | 1,803 | 370,340 |
| remove additional controls up to 28 days before a positive test | 1,422 | 368,918 |
| remove second episode where an individual has had A(H1N1) twice | 5 | 368,913 |
| remove second episode where an individual has had A(H3N2) twice | 5 | 368,908 |
| remove second episode where an individual has had flu B twice | 38 | 368,870 |
| remove flu A cases within 6 weeks | 112 | 368,758 |
| sample within 14 days of vaccination | 7,106 | 361,652 |
| remove if location unknown or outside PHE region | 38 | 361,614 |
| ineligible vaccine type | 139 | 361,475 |
| not hospitalised within -14 to 2 days of sample | 134,523 | 226,952 |
| remove control samples which are positive for covid | 16,050 | 210,902 |
| remove if hospitalisation is not ARI coded | 110,321 | 100,581 |

**Table S4** Exclusions for 2023-2024 data (SGSS and Datamart)

|  | n excluded | N in sample |
| --- | --- | --- |
| to start |  | 593,973 |
| ages 0 to 1 or no age | 4,890 | 589,083 |
| sample date before study start | 8 | 589,075 |
| sample date after study end | 42,425 | 546,650 |
| influenza status not fully known | 5,058 | 541,592 |
| de-duplication to 1 sample per person per day | 73,972 | 467,620 |
| remove control samples within 7 days of another sample | 78,615 | 389,005 |
| de-duplication to 1 control sample per person per 28-day period | 27,838 | 361,167 |
| de-duplication to 1 case sample per person per 28-day period | 3,652 | 357,515 |
| remove additional controls up to 28 days after a positive test | 1,243 | 356,272 |
| remove additional controls up to 28 days before a positive test | 1,502 | 354,770 |
| remove second episode where an individual has had A(H1N1) twice | 16 | 354,754 |
| remove second episode where an individual has had A(H3N2) twice | 7 | 354,747 |
| remove second episode where an individual has had flu B twice | 4 | 354,743 |
| remove flu A cases within 6 weeks | 37 | 354,706 |
| sample within 14 days of vaccination | 5,019 | 349,687 |
| remove if location unknown or outside PHE region | 0 | 349,687 |
| ineligible vaccine type | 110 | 349,577 |
| not hospitalised within -14 to 2 days of sample | 135,841 | 213,736 |
| remove control samples which are positive for covid | 2,864 | 210,872 |
| remove if hospitalisation is not ARI coded | 97,378 | 113,494 |

***Descriptive statistics***

**Table S5** Descriptive statistics for ages 2-17, 2022/23 season

**Table S6** Descriptive statistics for ages 18-64, 2022/23 season

**Table S7** Descriptive statistics for ages 65+, 2022/23 season

**Table S8** Descriptive statistics for ages 2-17, 2023/24 season

**Table S9** Descriptive statistics for ages 18-64, 2023/24 season

**Table S10** Descriptive statistics for ages 65+, 2023/24 season

***Additional figures and tables to illustrate seasonal trends in influenza and vaccination***

**Figure S1**. Percentage of influenza A(H1N1) or A(H3N2) detections by month, using data from four RDM laboratories that consistently subtype influenza A (includes non-hospitalised patients).

Influenza A(H3N2) dominated the 2022/23 season, and around 70% of detections were subtyped A(H3N2) from October 2022 through to February 2023. Influenza A detections during March-April 2023 were very low, but there were more influenza A(H1N1) than A(H3N2) detections during this period. The relative mix of A(H1N1) and A(H3N2) detections was fairly consistent throughout the 2023/24 season, with around 50-60% A(H1N1).

**Figure S2**. Percentage of vaccinations given by week and age group among vaccinated non-cases (includes non-hospitalised non-cases), overlaid on a count of hospitalised influenza positive cases.


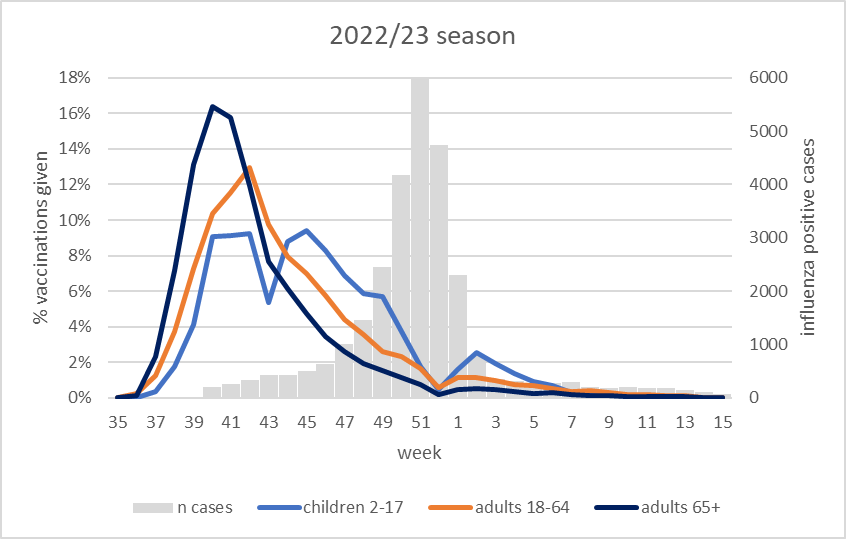


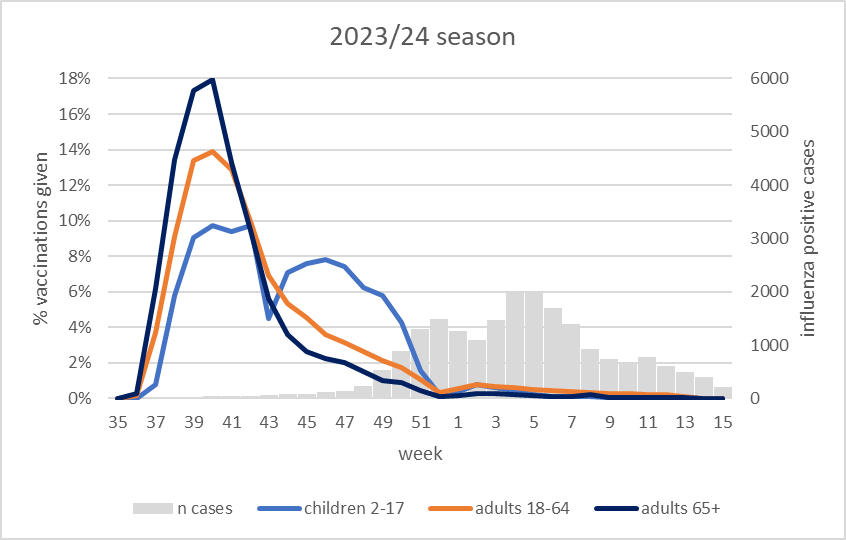


The above figure illustrates the timing of influenza vaccination against the background seasonal influenza hospitalisation. During the 2022/23 season the programme of influenza vaccination in schools continued into January 2023, after the peak of influenza in week 51 (late December 2022). During the 2023/24 season school immunisation teams aimed to cover all schools by December 2023. Elderly adults aged 65 years and older tend to receive vaccination earlier, on average, than other age groups.

**Figure S3. Time between vaccination and test (weeks) by vaccine type for each age cohort and season, all vaccinated cases and controls.**

**
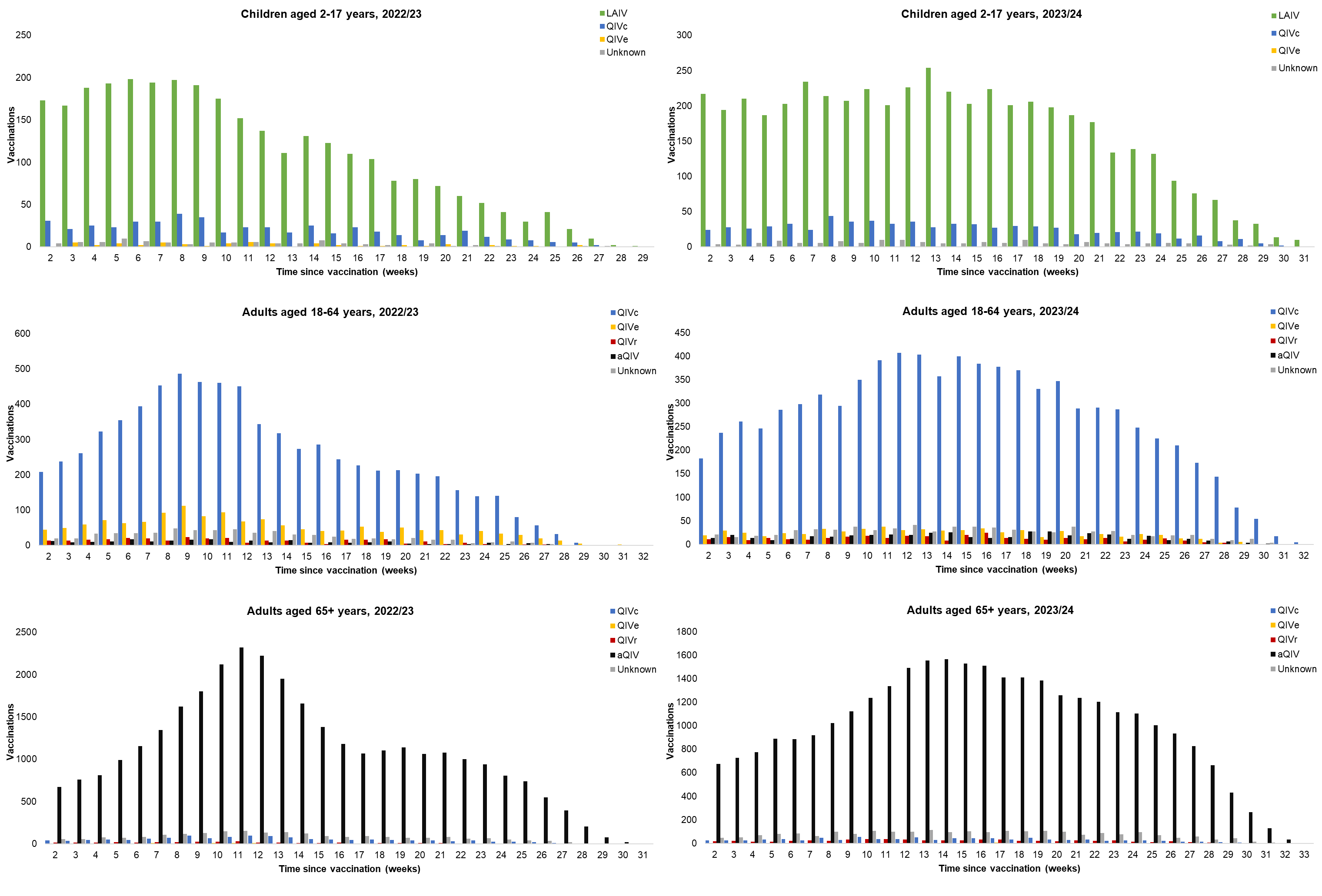
**

**Table S11. Descriptive table of age group and risk status by influenza vaccine type, for each age cohort and season. Count (n) with column percentage (%).**

|  |  |  |  | Unvaccinated | | LAIV | | QIVc | | QIVe | | QIVr | | aQIV | | Unknown | | (all vaccinated) | |
| --- | --- | --- | --- | --- | --- | --- | --- | --- | --- | --- | --- | --- | --- | --- | --- | --- | --- | --- | --- |
|  |  |  | | n | % | n | % | n | % | n | % | n | % | n | % | n | % | n | % |
| Children aged 2-17 2022/23 | Age group | 2 to 3 | | 3,336 | 33% | 983 | 32% | 123 | 25% | 20 | 36% | - | - | - | - | 26 | 28% | 1,152 | 31% |
|  |  | 4 to 6 | | 3,045 | 30% | 1,162 | 38% | 142 | 29% | 18 | 32% | - | - | - | - | 33 | 36% | 1,355 | 37% |
|  |  | 7 to 10 | | 1,662 | 16% | 611 | 20% | 116 | 24% | 5 | 9% | - | - | - | - | 15 | 16% | 747 | 20% |
|  |  | 11 to 15 | | 1,577 | 15% | 233 | 8% | 83 | 17% | 7 | 13% | - | - | - | - | 13 | 14% | 336 | 9% |
|  |  | 16 to 17 | | 598 | 6% | 43 | 1% | 29 | 6% | 6 | 11% | - | - | - | - | 5 | 5% | 83 | 2% |
|  | Risk group member | No | | 7,374 | 72% | 2,148 | 71% | 136 | 28% | 9 | 16% | - | - | - | - | 45 | 49% | 2,338 | 64% |
|  |  | Other than immunosuppressed | | 1,903 | 19% | 782 | 26% | 193 | 39% | 23 | 41% | - | - | - | - | 26 | 28% | 1,024 | 28% |
|  |  | Immunosuppressed | | 941 | 9% | 102 | 3% | 164 | 33% | 24 | 43% | - | - | - | - | 21 | 23% | 311 | 8% |
| Adults aged 18-64 2022/23 | Age group | 18 to 34 | | 6,582 | 31% | - | - | 915 | 13% | 163 | 11% | 36 | 12% | 10 | 4% | 105 | 16% | 1,229 | 12% |
|  |  | 35 to 49 | | 6,311 | 30% | - | - | 1,420 | 20% | 283 | 19% | 64 | 21% | 21 | 9% | 160 | 24% | 1,948 | 20% |
|  |  | 50 to 64 | | 8,367 | 39% | - | - | 4,885 | 68% | 1,015 | 69% | 206 | 67% | 212 | 87% | 399 | 60% | 6,717 | 68% |
|  | Risk group member | No | | 10,248 | 48% | - | - | 1,253 | 17% | 289 | 20% | 48 | 16% | 45 | 19% | 151 | 23% | 1,786 | 18% |
|  |  | Other than immunosuppressed | | 8,503 | 40% | - | - | 4,625 | 64% | 904 | 62% | 200 | 65% | 150 | 62% | 397 | 60% | 6,276 | 63% |
|  |  | Immunosuppressed | | 2,509 | 12% | - | - | 1,342 | 19% | 268 | 18% | 58 | 19% | 48 | 20% | 116 | 17% | 1,832 | 19% |
| Adults aged 65+ 2022/23 | Age group | 65 to 74 | | 6,506 | 34% | - | - | 598 | 43% | 79 | 57% | 118 | 33% | 8,453 | 26% | 598 | 26% | 9,846 | 27% |
|  |  | 75 to 84 | | 6,913 | 36% | - | - | 437 | 32% | 32 | 23% | 130 | 37% | 13,167 | 41% | 888 | 39% | 14,654 | 40% |
|  |  | 85+ | | 5,763 | 30% | - | - | 349 | 25% | 27 | 20% | 108 | 30% | 10,570 | 33% | 800 | 35% | 11,854 | 33% |
|  | Risk group member | No | | 3,199 | 17% | - | - | 193 | 14% | 23 | 17% | 40 | 11% | 4,374 | 14% | 316 | 14% | 4,946 | 14% |
|  |  | Other than immunosuppressed | | 13,537 | 71% | - | - | 977 | 71% | 95 | 69% | 268 | 75% | 23,245 | 72% | 1,687 | 74% | 26,272 | 72% |
|  |  | Immunosuppressed | | 2,446 | 13% | - | - | 214 | 15% | 20 | 14% | 48 | 13% | 4,571 | 14% | 283 | 12% | 5,136 | 14% |
| Children aged 2-17 2023/24 | Age group | 2 to 3 | | 3,491 | 35% | 1,740 | 35% | 181 | 25% | 1 | 25% | - | - | - | - | 24 | 15% | 1,946 | 34% |
|  |  | 4 to 6 | | 2,577 | 26% | 1,410 | 29% | 197 | 28% | 1 | 25% | - | - | - | - | 71 | 44% | 1,679 | 29% |
|  |  | 7 to 10 | | 1,706 | 17% | 1,119 | 23% | 142 | 20% | 1 | 25% | - | - | - | - | 34 | 21% | 1,296 | 22% |
|  |  | 11 to 15 | | 1,539 | 15% | 599 | 12% | 149 | 21% | 1 | 25% | - | - | - | - | 25 | 16% | 774 | 13% |
|  |  | 16 to 17 | | 720 | 7% | 56 | 1% | 42 | 6% | 0 | 0% | - | - | - | - | 6 | 4% | 104 | 2% |
|  | Risk group member | No | | 8,248 | 82% | 4,165 | 85% | 379 | 53% | 2 | 50% |  | - | - | - | 119 | 74% | 4,665 | 80% |
|  |  | Other than immunosuppressed | | 1,225 | 12% | 688 | 14% | 194 | 27% | 2 | 50% |  | - | - | - | 25 | 16% | 909 | 16% |
|  |  | Immunosuppressed | | 560 | 6% | 71 | 1% | 138 | 19% | 0 | 0% |  | - | - | - | 16 | 10% | 225 | 4% |
| Adults aged 18-64 2023/24 | Age group | 18 to 34 | | 8,072 | 30% | - | - | 1,146 | 14% | 98 | 15% | 55 | 16% | 15 | 3% | 149 | 20% | 1,463 | 14% |
|  |  | 35 to 49 | | 8,139 | 30% | - | - | 1,848 | 22% | 132 | 20% | 82 | 24% | 26 | 6% | 164 | 22% | 2,252 | 22% |
|  |  | 50 to 64 | | 11,027 | 40% | - | - | 5,266 | 64% | 422 | 65% | 203 | 60% | 425 | 91% | 427 | 58% | 6,743 | 64% |
|  | Risk group member | No | | 19,634 | 72% | - | - | 4,218 | 51% | 344 | 53% | 178 | 52% | 242 | 52% | 377 | 51% | 5,359 | 51% |
|  |  | Other than immunosuppressed | | 5,833 | 21% | - | - | 3,104 | 38% | 244 | 37% | 123 | 36% | 166 | 36% | 298 | 40% | 3,935 | 38% |
|  |  | Immunosuppressed | | 1,771 | 7% | - | - | 938 | 11% | 64 | 10% | 39 | 11% | 58 | 12% | 65 | 9% | 1,164 | 11% |
| Adults aged 65+ 2023/24 | Age group | 65 to 74 | | 8,138 | 33% | - | - | 310 | 35% | 17 | 39% | 157 | 27% | 9,161 | 29% | 554 | 24% | 10,199 | 29% |
|  |  | 75 to 84 | | 9,107 | 37% | - | - | 342 | 39% | 20 | 45% | 242 | 41% | 13,137 | 42% | 954 | 42% | 14,695 | 41% |
|  |  | 85+ | | 7,288 | 30% | - | - | 228 | 26% | 7 | 16% | 190 | 32% | 9,333 | 30% | 781 | 34% | 10,539 | 30% |
|  | Risk group member | No | | 12,694 | 52% | - | - | 439 | 50% | 28 | 64% | 326 | 55% | 16,296 | 52% | 1,181 | 52% | 18,270 | 52% |
|  |  | Other than immunosuppressed | | 9,739 | 40% | - | - | 348 | 40% | 13 | 30% | 220 | 37% | 12,494 | 39% | 958 | 42% | 14,033 | 40% |
|  |  | Immunosuppressed | | 2,100 | 9% | - | - | 93 | 11% | 3 | 7% | 43 | 7% | 2,841 | 9% | 150 | 7% | 3,130 | 9% |

***Vaccine effectiveness using Respiratory DataMart (RDM) data alone***

**Figure S4** Vaccine effectiveness by broad age group, for all influenzas and by influenza type/subtype: influenza A (all subtypes), A(H1N1), A(H3N2), influenza B, based on DataMart data alone.


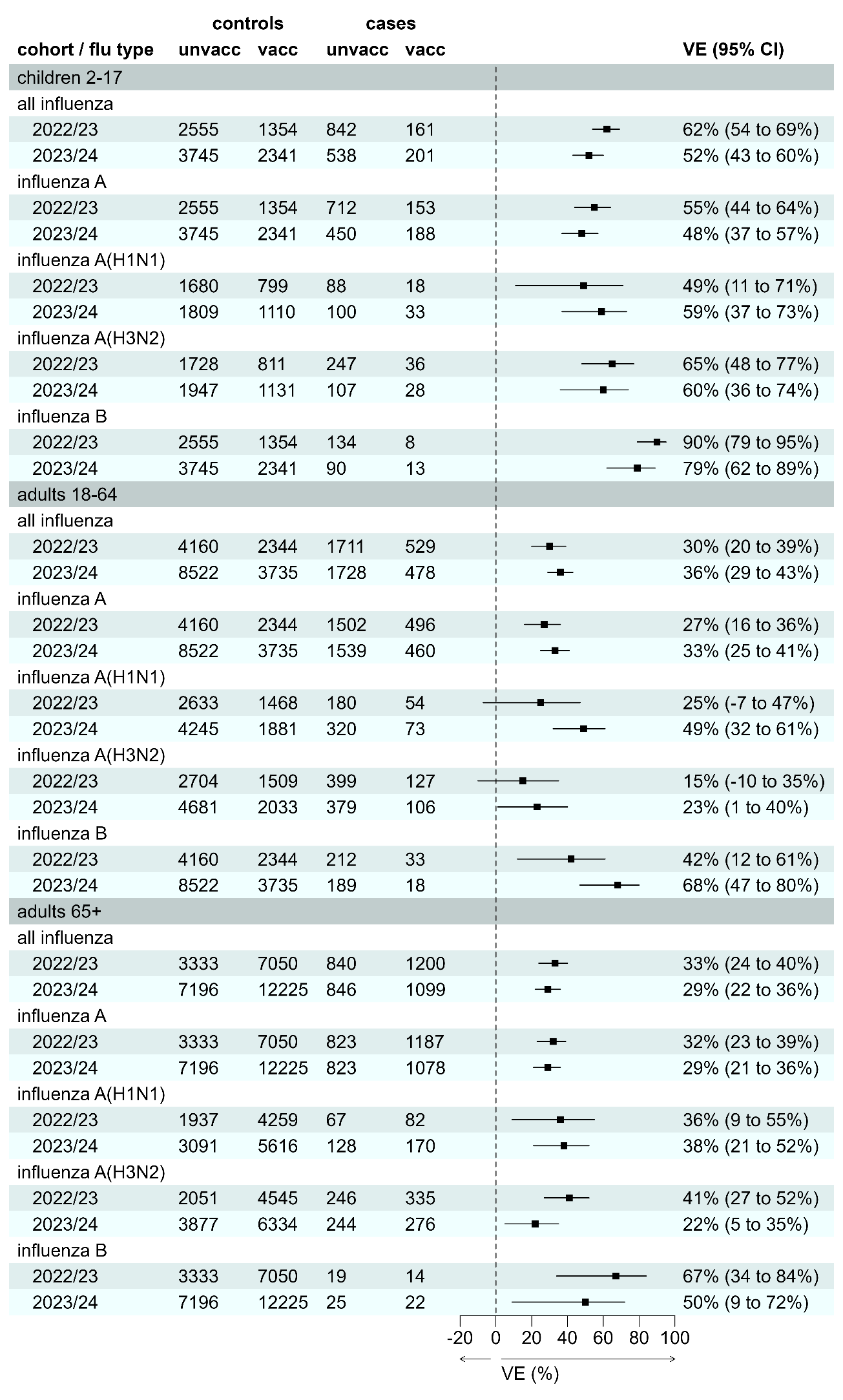


***Self-controlled case series (SCCS) for waning relative VE (rVE)***

**Box S1 Additional information and methods on self controlled case series analyses for waning relative VE**

The SCCS design was additionally used to explore waning VE relative to the first period (2-8 weeks) post vaccination. This case-only design (i.e. using only positive tests) can be used where timing of events is key, as for analyses of waning.

Use of SCCS for vaccine effectiveness in general is complicated by behaviour. Assumptions of the method fail when vaccination does not take place (or conversely does take place) specifically because a patient tested positive for influenza, which leads to bias; these behaviours are not sufficiently well understood to enable accurate bias correction. However, analyses of waning rVE can focus on post-vaccination time only, removing this issue.

A benefit of SCCS is that VE should not be biased by test sensitivity (assuming that this does not change with time since vaccination), which is useful given missing data on onset date (sensitivity declines with time since onset); false negative tests will only lead to loss of power in SCCS. In a TND false negatives will bias VE toward the null, but relative VE should be unbiased. An additional benefit is that all confounders that can reasonably be assumed to be fixed over the period of one season factor out of a self-controlled model completely; this includes health seeking behaviours and underlying frailty.

Limitations of SCCS include susceptibility to bias due to depletion of susceptibles (see main paper discussion). Good estimation of the change in influenza incidence over the season is key to the SCCS design, and this may not have been achieved where the overall number of cases was low.

In fitting the SCCS model, we included for vaccinated cases all study time from 2-weeks post vaccination until the study end used in TND analyses, plus all study time in unvaccinated cases (who were included to help estimate influenza incidence over time, they do not otherwise contribute to vaccine effects). As for the TND, analyses were run separately for children, working age and elderly adults. We used the same categories for time since vaccination (2-8, 9-12, 13-16 and 17+ weeks). Season was adjusted for by including a week effect, but aggregating two-week periods where case numbers were low at the beginning or end of the season.

Results of both the TND and SCCS are given in Figures S5 and S6. rVE estimates were broadly similar between the test negative and self-controlled case series (SCCS) designs, and overall conclusions unaltered. Waning in adults against influenza A was a little less steep using the SCCS design.

**References**

Farrington P, Whitaker H, Ghebremichael Weldeselassie Y. Self-Controlled Case Series Studies: A Modelling Guide with R. Chapman and Hall/CRC, New York, 2018. https://doi.org/10.1201/9780429491313

Whitaker HJ, Ghebremichael-Weldeselassie Y, Douglas IJ, Smeeth L, Farrington CP. Investigating the assumptions of the self-controlled case series method. Stat Med. 2018 Feb 20;37(4):643-658. https://doi.org/10.1002/sim.7536

**Figure S5** Waning vaccine effectiveness: Comparison of test negative and SCCS, 2022/23 season.


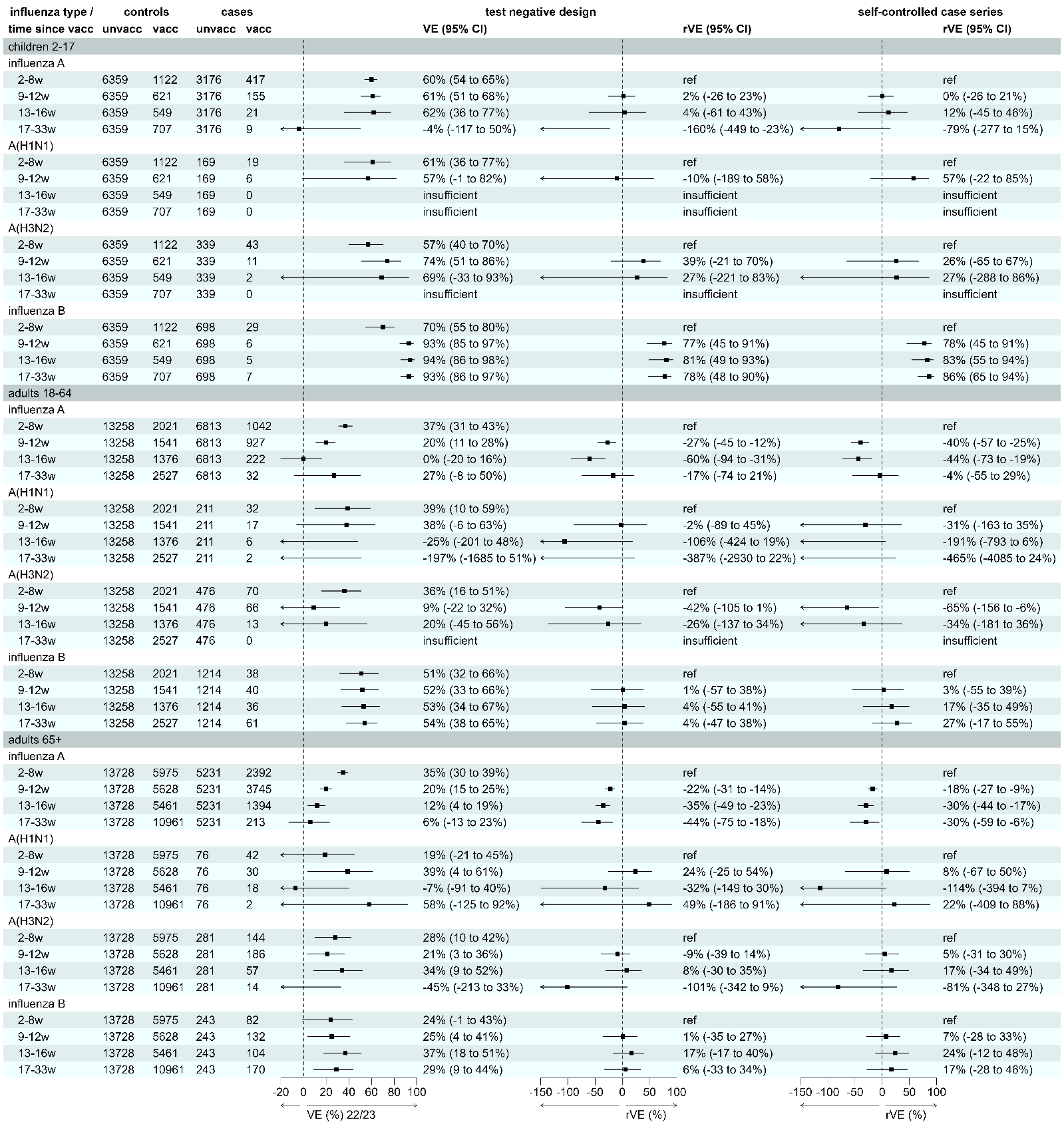


VE = vaccine effectiveness, rVE = relative vaccine effectiveness (relative to 2-8weeks post vaccination)

**Figure S6** Waning vaccine effectiveness: Comparison of test negative and SCCS, 2023/24 season.


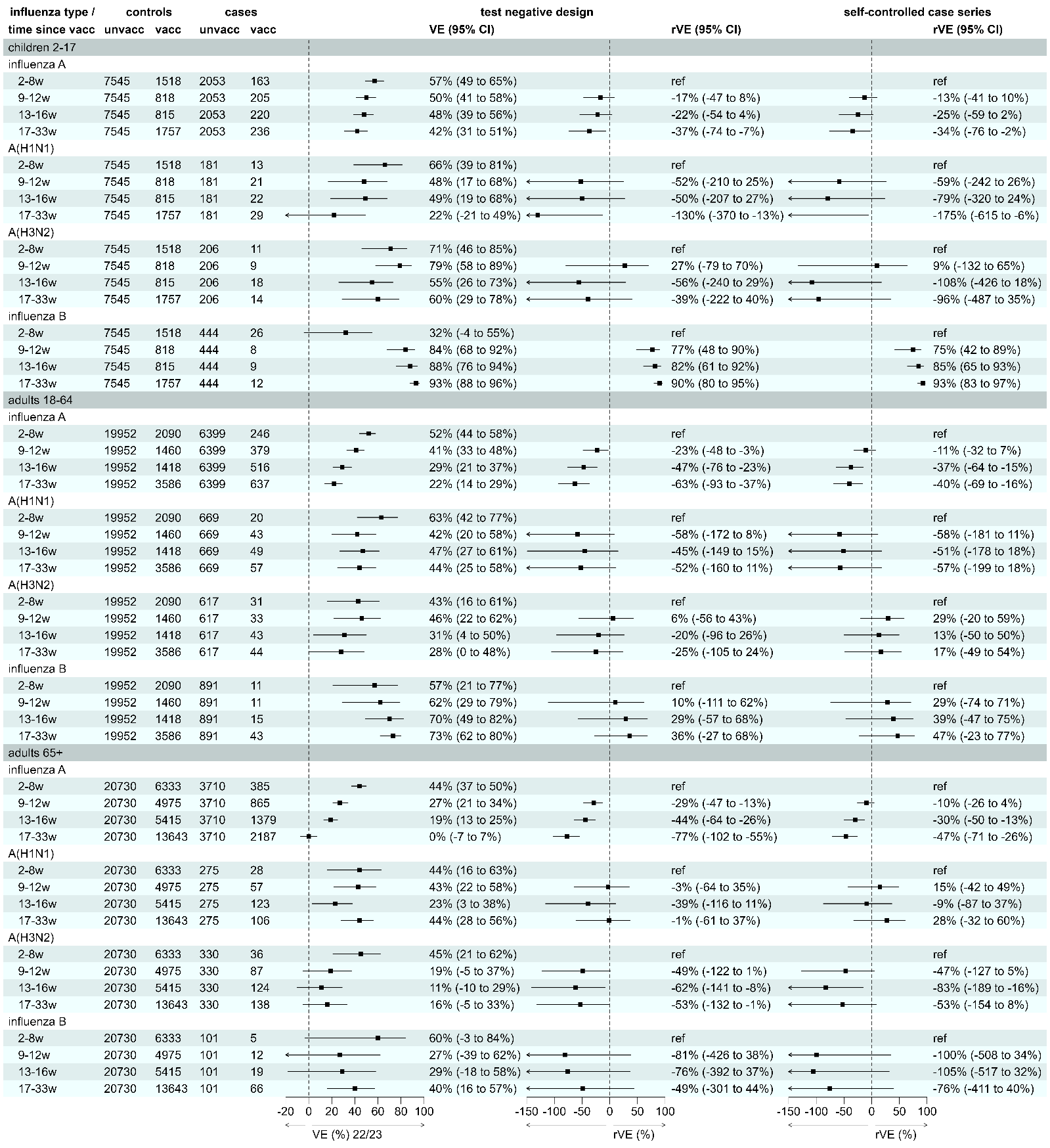


VE = vaccine effectiveness, rVE = relative vaccine effectiveness (relative to the 2-8week post vaccination period)

***Sensitivity analyses for waning VE/rVE***

**Figure S7** Waning vaccine effectiveness: Original analysis (upper) and sensitivity analysis (lower) in Children aged 2-17 that excludes week 2 post vaccination (the reference category for rVE is 3-8weeks)


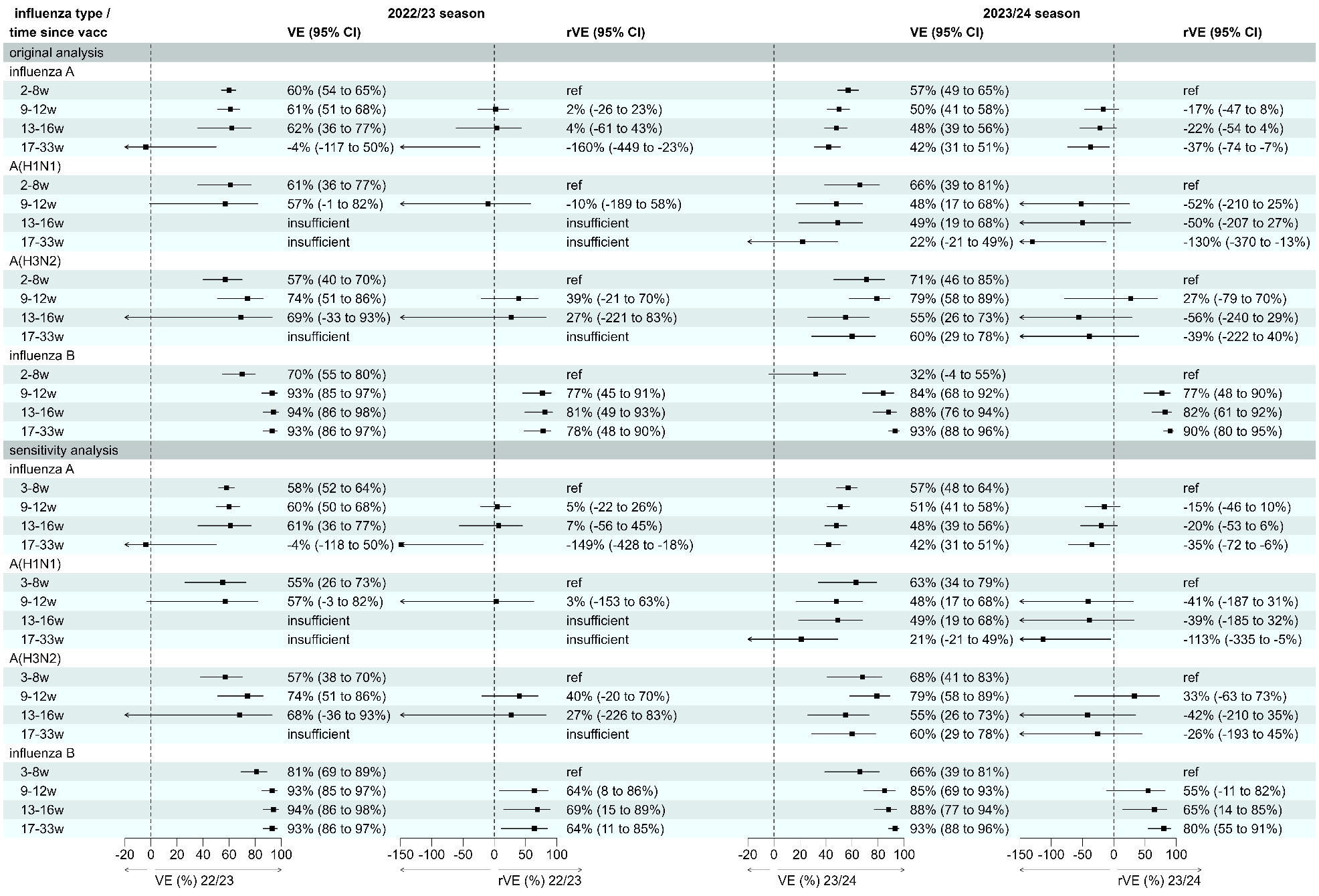
It is possible that LAIV detections may extend beyond the 0-13 days post vaccination that is excluded from the original analysis. The sensitivity analysis extends this exclusion period to 0-20 days for children aged 2-17, to ensure LAIV detections are eliminated. Estimates for all influenza A are little changed by the additional exclusion. However, VE for influenza B during the period 3-8weeks post vaccination (sensitivity analysis) are higher than VE estimates for the period 2-8weeks post vaccination (original analysis). While rVE is reduced in the influenza B sensitivity analysis, estimates remain positive.

VE = vaccine effectiveness, rVE = relative vaccine effectiveness (relative to the first [2-8 or 3-8 week] post-vaccination period)

**Figure S8** Waning vaccine effectiveness: two categories


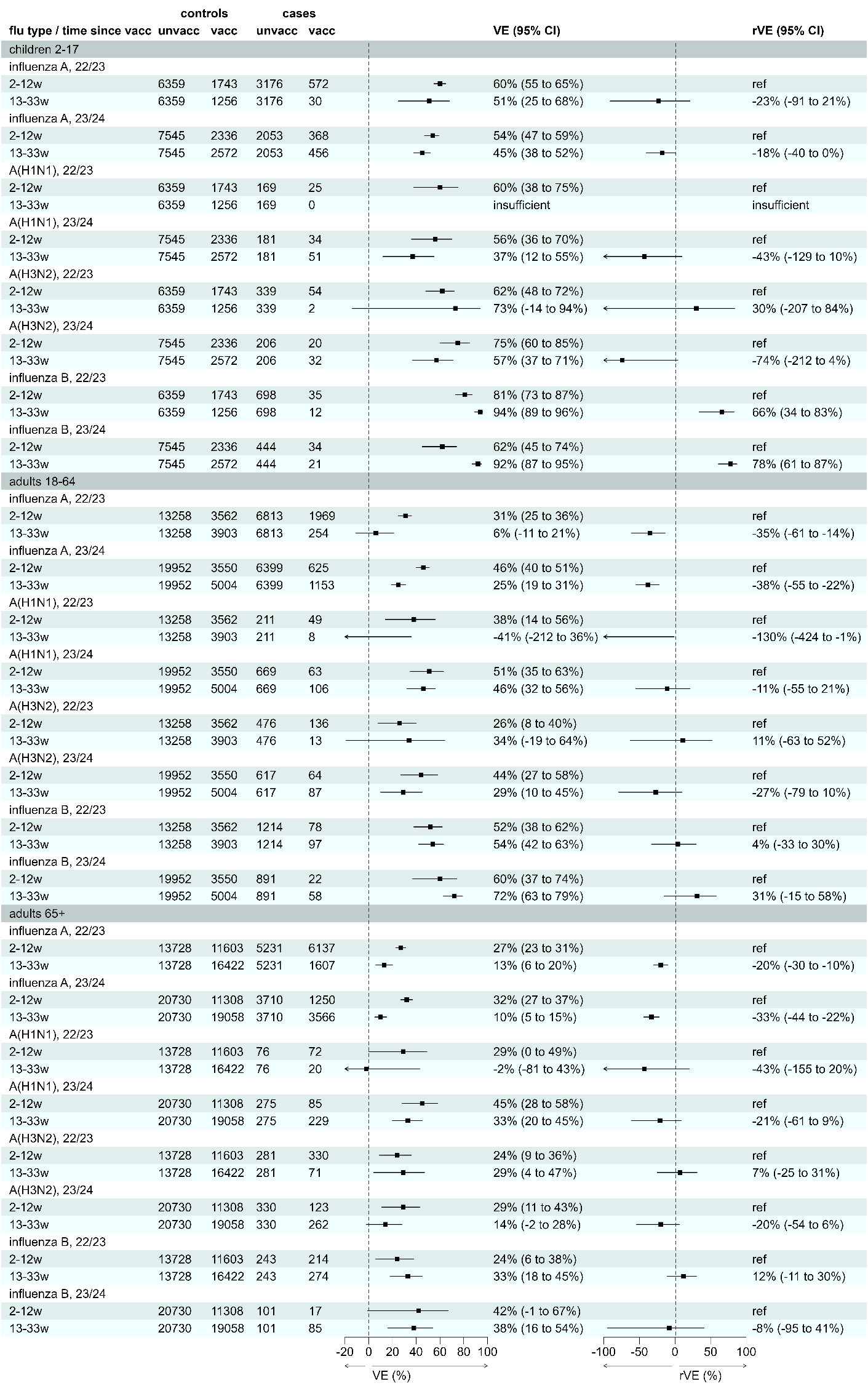


VE = vaccine effectiveness, rVE = relative vaccine effectiveness (relative to 2-12weeks post vaccination)

**Figure S9** Waning vaccine effectiveness 2022/23 season: original analysis and sensitivity analysis including only those vaccinated by 7 November 2022 (when influenza activity began to rise)


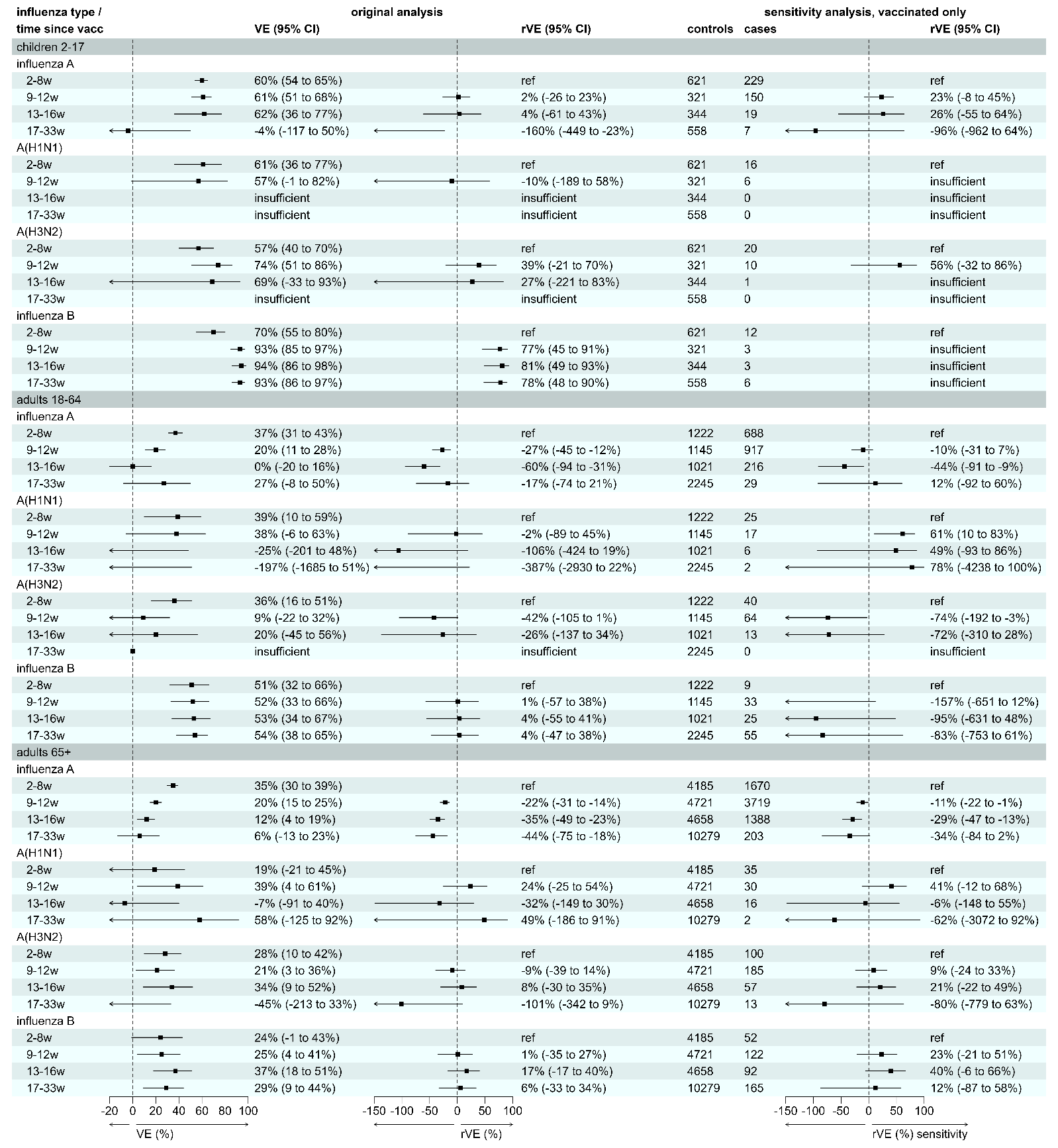


VE = vaccine effectiveness, rVE = relative vaccine effectiveness (relative to 2-8weeks post vaccination)

**Figure S10** Waning vaccine effectiveness 2023/24 season: original analysis and sensitivity analysis including only those vaccinated by 4 December 2023 (when influenza activity began to rise)


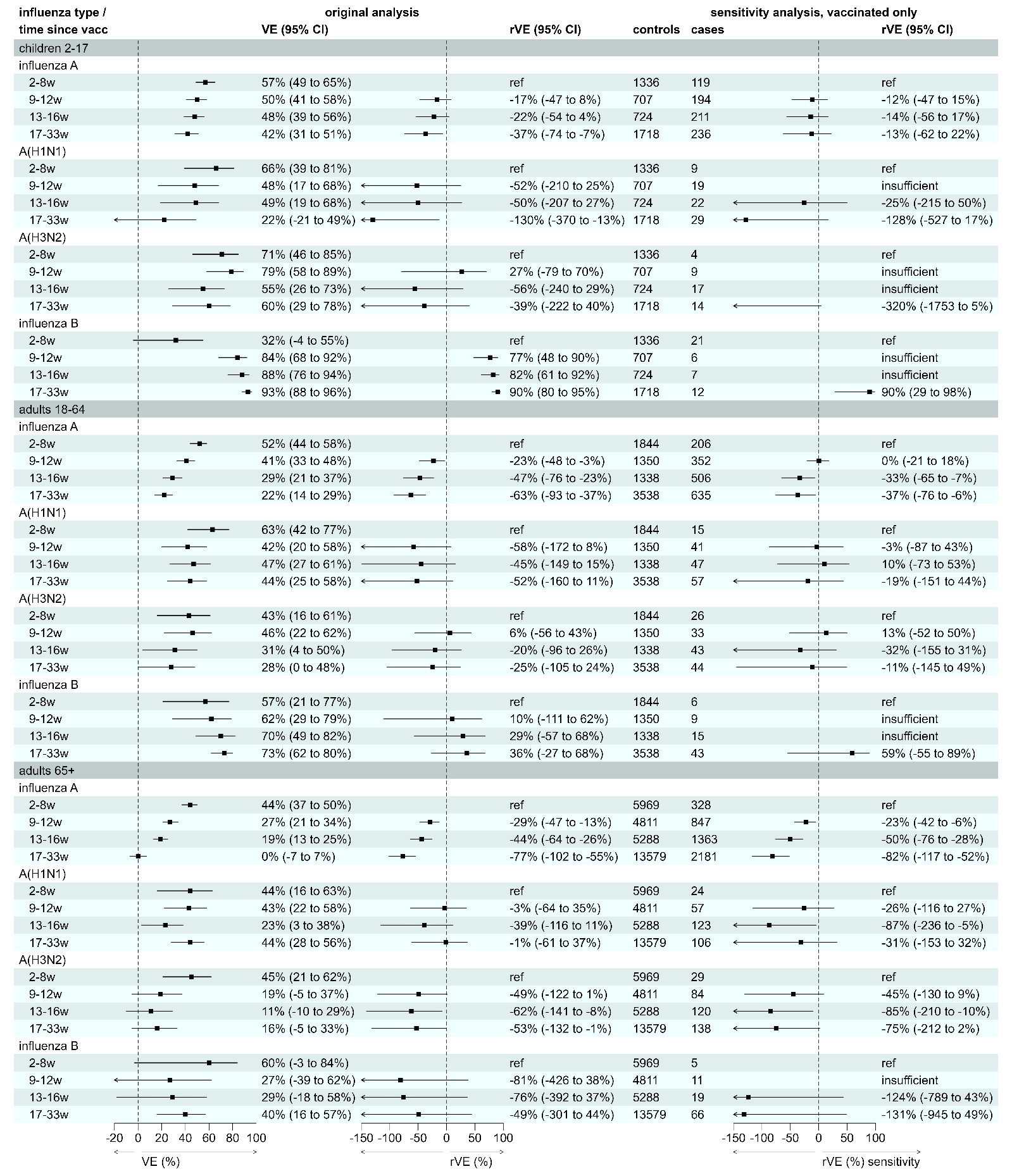


VE = vaccine effectiveness, rVE = relative vaccine effectiveness (relative to 2-8weeks post vaccination)

**Current and past season vaccination. Figure S11** Combined current and past season vaccine effectiveness.


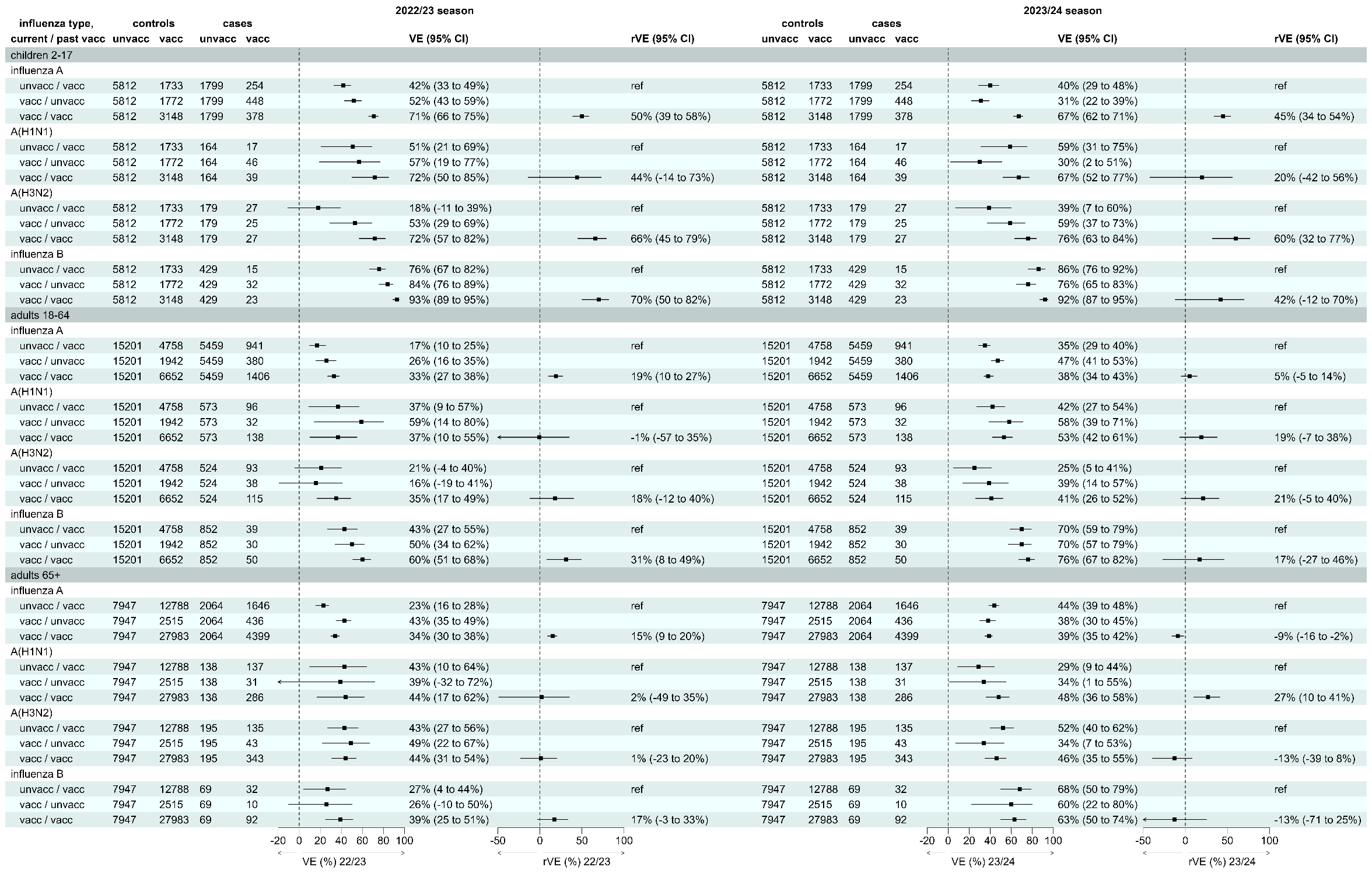


VE = vaccine effectiveness (relative to unvaccinated both seasons), rVE = relative vaccine effectiveness (relative to unvaccinated in the current season and vaccinated in the past season).

unvacc / vacc = unvaccinated in the current season and vaccinated in the past season, vacc / unvacc = vaccinated in the current season and unvaccinated in the past season, vacc / vacc = vaccinated both seasons.
